# Human Placental Trophoblasts Infected by *Listeria monocytogenes* Undergo a Pro-Inflammatory Switch Associated With Poor Pregnancy Outcomes

**DOI:** 10.3389/fimmu.2021.709466

**Published:** 2021-07-23

**Authors:** Lauren J. Johnson, Siavash Azari, Amy Webb, Xiaoli Zhang, Mikhail A. Gavrilin, Joanna M. Marshall, Kara Rood, Stephanie Seveau

**Affiliations:** ^1^ Department of Microbial Infection and Immunity, The Ohio State University, Columbus, OH, United States; ^2^ Department of Microbiology, The Ohio State University, Columbus, OH, United States; ^3^ Department of Biomedical Informatics, The Ohio State University, Columbus, OH, United States; ^4^ Department of Biomedical Informatics, Center for Biostatistics, The Ohio State University, Columbus, OH, United States; ^5^ Pulmonary, Critical Care and Sleep Medicine Division, Department of Internal Medicine, The Ohio State University, Columbus, OH, United States; ^6^ Department of Obstetrics and Gynecology, Division of Maternal Fetal Medicine, The Ohio State University, Columbus, OH, United States; ^7^ Infectious Diseases Institute, The Ohio State University, Columbus, OH, United States

**Keywords:** trophoblast, fusion, placenta, *Listeria monocytogenes*, infection, inflammation, RNAseq, pregnancy complications

## Abstract

The placenta controls the growth of the fetus and ensures its immune protection. Key to these functions, the syncytiotrophoblast (SYN) is a syncytium formed by fusion of underlying mononuclear trophoblasts. The SYN covers the placental surface and is bathed in maternal blood to mediate nutritional and waste exchanges between the mother and fetus. The bacterial pathogen *Listeria monocytogenes* breaches the trophoblast barrier and infects the placental/fetal unit resulting in poor pregnancy outcomes. In this work, we analyzed the *L. monocytogenes* intracellular lifecycle in primary human trophoblasts. In accordance with previous studies, we found that the SYN is 20-fold more resistant to infection compared to mononuclear trophoblasts, forming a protective barrier to infection at the maternal interface. We show for the first time that this is due to a significant reduction in *L. monocytogenes* uptake by the SYN rather than inhibition of the bacterial intracellular division or motility. We here report the first transcriptomic analysis of *L. monocytogenes*-infected trophoblasts (RNA sequencing). Pathway analysis showed that infection upregulated TLR2, NOD-like, and cytosolic DNA sensing pathways, as well as downstream pro-inflammatory circuitry (NF-κB, AP-1, IRF4, IRF7) leading to the production of mediators known to elicit the recruitment and activation of maternal leukocytes (IL8, IL6, TNFα, MIP-1). Signature genes associated with poor pregnancy outcomes were also upregulated upon infection. Measuring the release of 54 inflammatory mediators confirmed the transcriptomic data and revealed sustained production of tolerogenic factors (IL-27, IL-10, IL-1RA, TSLP) despite infection. Both the SYN and mononuclear trophoblasts produced cytokines, but surprisingly, some cytokines were predominantly produced by the SYN (IL-8, IL-6) or by non-fused trophoblasts (TNFα). Collectively, our data support that trophoblasts act as placental gatekeepers that limit and detect *L. monocytogenes* infection resulting in a pro-inflammatory response, which may contribute to the poor pregnancy outcomes if the pathogen persists.

## Introduction

*Listeria monocytogenes* is a foodborne pathogen that primarily affects the elderly, pregnant women, and immuno-compromised individuals. During pregnancy, the mother generally experiences mild symptoms, but vertical transmission of *L. monocytogenes* occurs *via* breaching the placental barrier leading to fetal death, preterm birth, and severe infections of the newborn (1–7). Several studies support that *L. monocytogenes* enters the placenta *via* infection of epithelial cells that delineate the maternal/fetal interface: the syncytiotrophoblast (SYN) and extravillous trophoblasts (8–11). We studied the interplay between primary human trophoblasts, isolated from term pregnancies, and *L. monocytogenes* to gain understanding of their responses to infection and their potential role in poor pregnancy outcomes.

The placenta is an essential organ that nourishes, protects, and supports the growth of the fetus. Central to these functions, trophoblasts are epithelial cells derived from the outer layer of the blastocyst (trophectoderm) that play key roles in blastocyst implantation in the maternal uterine tissue, in the production of pregnancy hormones, and in placentation. Trophoblasts play essential roles in controlling fetal development and immunity (12). Various trophoblast subtypes specialize in distinct functions. The syncytiotrophoblast (SYN), formed by fusion of underlying mononuclear trophoblasts, is a syncytium that covers the ~12 m^2^ surface of chorionic villi and is bathed in maternal blood to allow all nutritional and waste exchanges between mother and fetus (3, 13–16). The SYN also secretes pregnancy hormones and releases extracellular vesicles in maternal blood (17, 18). A second category of trophoblasts, the extravillous trophoblasts, are located at the tip of placental villi, forming columns of mononuclear cells that anchor villi into the maternal endometrium (12). A third type of trophoblasts, the endovascular trophoblasts, which differentiate from extravillous trophoblasts, remodel the maternal spiral arteries that provide the placenta with maternal blood (12). Due to their location at the interface with maternal cells, trophoblasts play critical roles in promoting immune tolerance of the semi-allogeneic fetal cells *via* controlling expression of immune modulatory surface receptors and releasing tolerogenic mediators (19–21).

The SYN has been shown to be less permissive to infection compared to mononuclear trophoblasts, but the underlying mechanisms are not fully elucidated (3, 22). Although the SYN, and other trophoblasts form an effective barrier to infection, some pathogens, such as *Toxoplasma gondii*, Zika virus, herpes simplex virus, cytomegalovirus, and *L. monocytogenes*, can breach this cell barrier with devastating consequences for the developing fetus (16, 22–30). The deleterious effects of infection are not solely due to the presence of the pathogen in the placental-fetal unit, but also derive from its consequential inflammation. Inflammation is a normal manifestation of the anti-infective host responses aimed at eliminating the pathogen, but placental inflammation can be particularly deleterious. Indeed, excessive placental inflammation (placentitis, placental villitis, and chorioamnionitis) causes preterm birth by mimicking the rise of inflammatory mediators involved in labor and parturition (31–34). Also, misguided placental inflammation can disrupt the immunological tolerance of the semi-allogeneic fetus leading to fetal rejection and abortion (35). Therefore, establishing the trophoblast inflammatory responses to infectious agents and their potential contribution to placental immunity and inflammation is of paramount importance.

*L. monocytogenes* is a facultative intracellular pathogen that infects professional phagocytes and normally non-phagocytic cells, such as epithelial cells (8). In accordance with the notion that placental pathogens have an intracellular lifestyle, *L. monocytogenes* virulence factors involved in host cell invasion are also critical for placental infection, as demonstrated using small animal models (36–40). These virulence factors include the surface proteins InlA and InlB, which facilitate *L. monocytogenes* internalization into cells that express their receptors: the adherens junction protein E-cadherin and the HGF-receptor c-Met, respectively (8, 41, 42). The secreted pore-forming toxin listeriolysin O (LLO) and the surface protein ActA, are respectively critical for bacterial escape from the phagosome and cell-to-cell spreading. However, LLO and ActA are multifunctional as they both also promote bacterial internalization in some, but not all, host cells (43–45). Several placenta-specific virulence factors were identified including InlP, which is a secreted factor thought to play a role in *L. monocytogenes* crossing of the basement membrane of chorionic villi (46, 47). Placental infection by *L. monocytogenes* is accompanied by placental inflammation, as observed from clinical cases and animal studies (5, 7, 48–50). However, the cell types and mechanisms contributing to inflammation in the placenta have yet to be determined.

In the present work, we isolated primary human trophoblasts from healthy term placentas. These cells spontaneously fuse in cell culture, mimicking the SYN. We also used the human choriocarcinoma BeWo cell line that can fuse upon activation of adenylate cyclase by forskolin, as a surrogate trophoblast model (51, 52). We characterized the *L. monocytogenes* intracellular lifecycle in syncytia (fused) and non-fused trophoblasts to establish molecular mechanisms underlying SYN resistance to infection. RNA sequencing (RNAseq) of *L. monocytogenes* infected trophoblasts in conjunction with monitoring cellular production of 54 inflammatory mediators established the trophoblast pro-inflammatory circuitry in response to *L. monocytogenes* infection.

## Materials and Methods

### Human Cell Culture, Isolation, Purity, and Fusion Efficiency

*Primary human trophoblasts (PHT)* were isolated from healthy, singleton, term placentas collected by Caesarean section (OSU Wexner Medical Center, Columbus, Ohio) according to the Institutional Review Board protocol # 2017H0478. A total of 24 placentas were used in the presented studies. Tissue dissection, enzymatic digestion and cell separation were performed as previously described (53). Collected cells were plated at 10^6^ cells/1.9 cm^2^ (with glass coverslip for microscopy experiments) washed after 12 h to remove non-adherent cells, and cultured in Dulbecco’s modified Eagle’s medium containing F12 (DMEM/F12 Gibco), GlutaMAX, 10% heat-inactivated fetal bovine serum (HI-FBS, Atlanta Biologicals), 100 U/ml penicillin, and 100 μg/ml streptomycin (Invitrogen) at 37°C in 5% CO_2_ humidified cell culture incubator. *Cell purity* was 95 ± 0.9% (n=8) (**Figure S1A**), as measured by fluorescence microscopy after 72 h of culture, which accounts for proliferation of contaminating fibroblasts at the time of all infection procedures carried out in this study. This method was preferred over flow cytometry that measures fluorescence intensities of detached, suspended cells. Indeed, microscopy allows measuring purity of attached cells, avoiding detaching strongly adherent cells and large syncytia, which would have caused major cell damage. For all fluorescence labeling, cells were fixed with 4% paraformaldehyde (PFA) in phosphate buffered saline (PBS) pH 7.4 for 20 min at room temperature (RT) and blocked for 1 h in PBS, 0.1 M glycine and 10% HI-FBS. For purity measurement, cells were co-labeled with primary mouse anti-cytokeratin 7 (Abcam, OV-TL 12/30, 1:100), rabbit anti-vimentin (Abcam, ab16700, 1:200), and secondary antibodies conjugated to Alexa Fluor dyes (Molecular Probes). Coverslips were mounted in Prolong Diamond Antifade (Molecular Probes) containing DAPI to label nuclei. Ten sets of phase-contrast and fluorescence images were randomly acquired, in duplicate, with a 40X objective, corresponding to at least 2200 cells (nuclei) per experiment. Purity was enumerated by counting the number of nuclei in cytokeratin 7 positive cells (trophoblasts, N_c_) and in vimentin positive cells (macrophages and fibroblasts, N_v_). Trophoblast purity was calculated as follows: [N_c_/(N_c_ + N_v_)] x 100. Human chorionic gonadotropin (β-hCG) from cell culture supernatants was measured by ELISA (DRG international) as per manufacturers’ instructions (**Figure S1B**). *Syncytia formation* (cell fusion) was also measured by fluorescence microscopy, by fluorescence labeling with mouse anti-E-cadherin (Abcam, HECD-1, 1:100), rabbit anti-cytokeratin 7 (Abcam, ab53123, 1:300), followed by labeling with Alexa Fluor-conjugated secondary antibodies, Alexa Fluor 488-Phalloidin, and DAPI (15, 54). A minimum of 2000 nuclei were counted per experiment and the percentage of cell fusion was calculated based on the number of nuclei in fused (N_f_) and non-fused trophoblasts (N_nf_) as follows: [N_f/_(N_f_ + N_nf_)] x 100. Only cells containing a minimum of three nuclei were considered to be fused. *The human choriocarcinoma cell line BeWo* (ATCC CCL-98) was a kind gift from Dr. John Mitchell Robinson (The Ohio State University, OH, USA). BeWo cells were mycoplasma-free and were authenticated by ATCC by short tandem repeat DNA profiling. BeWo cells were plated in DMEM/F12 supplemented with GlutaMAX plus 10% HI-FBS and penicillin/streptomycin at 5 x 10^4^ cells/cm^2^ for fused, and 2.5 x 10^4^ cells/cm^2^ for non-fused experimental conditions (55). Fusion was induced after 24 h of culture by daily addition of 20 µM forskolin (FSK, Sigma-Aldrich) for up to 72 h. Cell fusion was measured by β-hCG quantification (**Figure S1B**) by ELISA and by fluorescence microscopy (as described for PHT) at the following time points: 0 (after 24 h of culture), 24, 48, and 72 h after addition of FSK. *HeLa cells* (ATCC CCL-2) were mycoplasma-free and authenticated by ATCC, *via* short tandem repeat DNA profiling. They were plated in 24-well plates at 2x10^5^ cells/well 24 h prior to infection in DMEM supplemented with 10% HI-FBS, 100 U/ml penicillin and 100 μg/ml streptomycin at 37°C in 5% CO_2_ humidified cell culture incubator.

### *Listeria monocytogenes* Strains and Culture

Wild-type (WT) *L. monocytogenes* (10403S) and isogenic Δ*inlA* (DP-L4405), Δ*inlB* (DP10403S), Δ*hly* (DP-L2161), Δ*actA* (DP-L3078) mutants were gifts from Dr. Daniel Portnoy (UC Berkeley, CA, USA). The isogenic p*actA*-RFP, which expresses the red fluorescent protein under the control of the *actA* promoter, was a gift from Dr. Anna I. Bakardjiev (UC San Francisco, CA, USA) (11). *L. monocytogenes* 10403S, a streptomycin-resistant derivative of strain 10403 (lineage II and serotype 1/2a), was originally isolated from a human skin lesion (56) (57, 58). For invasion assays, bacteria were grown overnight under agitation at 37°C in brain heart infusion broth (BHI; BD Biosciences) until OD_600_ of 0.7-0.9 was reached. Bacteria were washed three times at 37°C and suspended in cell culture medium without serum and antibiotics for infection experiments.

### Fluorescence Microscopy Analysis of Trophoblast Invasion by*L. monocytogenes*

PHT and BeWo, cultured in 24-well plates, were infected in duplicate with 10^6^ *listeria monocytogenes (Lm)*/well. Cell culture plates were centrifuged at RT for 2 min at 1500 x *g*, incubated 1 h at 37°C, then washed three times and incubated with DMEM/F12, 10% HI-FBS and 15 μg/ml gentamicin for 1 h at 37°C. For bacterial labeling, extracellular and total bacteria were labeled with rabbit anti-*Lm* (GeneTex, 1:100) and anti-rabbit Alexa Fluor-conjugated secondary antibodies as previously described (59). Briefly, extracellular bacteria were first labeled on non-permeabilized cells with a first fluorochrome; whereas, total bacteria (extracellular plus intracellular) were labeled with a distinct fluorochrome following cell permeabilization with 0.3% Triton-X100. E-cadherin and F-actin (Alexa Fluor-phalloidin, Molecular Probes) labeling were performed in parallel to distinguish fused from non-fused cells. Ten sets of phase-contrast and fluorescence images were randomly acquired, in duplicate (20 image sets) for each experimental condition using a 100X objective. The numbers of total bacteria (N_t_), extracellular bacteria (N_e_), and DAPI-labeled nuclei (N_n_) were enumerated in fused and non-fused cells. Bacterial association efficiency was calculated as N_t_/N_n_. The internalization efficiency was calculated as 100 x (N_t_ – N_e_)/N_t_. Typically, 1000 - 2500 host cell nuclei were counted for each experimental condition.

### Live-Cell Fluorescence Microscopic Analysis of *L. monocytogenes* Intracellular Division Time and Motility

Trophoblasts were plated at 2x10^6^ cells per 35 mm diameter imaging dish (Ibidi) and cultured at 37°C in 5% CO_2_ for 72 h. Cells were infected with *p*actA-*L. monocytogenes* at MOI of 1 for a total of 5 h (1 h infection, followed by 30 min treatment with 15 µg/ml gentamicin, and 3.5 h incubation in cell culture medium without antibiotics) then washed 3 times with PBS at 37°C. Cells were incubated at 37°C with 0.1X CellMask Deep Red (Invitrogen) plasma membrane dye and Hoechst 33342 (Thermo Scientific) at 0.1 µg/ml for 5 min in cell culture medium without phenol red and supplemented with L-glutamine and HEPES pH 7.4 (imaging medium). Cells were washed 3x with warm PBS and incubated in imaging medium on the microscope stage at 37°C. Fluorescent images were acquired as Z-stacks with the 100X objective every 5 sec for 5 min to measure bacterial motility. Bacteria were tracked through each plane of the Z-stack using the multi-line function of MetaMorph (Molecular Devices). Each plane is considered 5 sec and the total µm/min was calculated based on the total length of each bacterial track through the Z-stack.

To determine 2 h division time, infected areas were chosen with priority given to fused areas. Initial phase-contrast, DSRED (*L. monocytogenes*), CY5 (plasma membrane), and DAPI images were taken for at least four positions to make overlays. Z-stacks of each position in the DSRED color channel were taken at 5 h, 6 h and 7 h. The 2 hour division time was calculated based on the difference between the initial and final DSRED images using the following equations: growth rate (gr) = (ln [N(t)/N(0)])/t and doubling time = ln (2)/gr, where N(t) is the final number of bacteria, N(0) is the initial number of bacteria, and t is the time point in hours.

### TLR2 Analyses

*TLR2 labeling* was performed on fixed and permeabilized cells using rabbit anti-TLR2 (Invitrogen, clone JM22-41, 1:100) primary antibodies. *TLR2 western blot*: 25 µg of protein from cell lysates of infected and non-infected PHT were loaded on a 10% tris-glycine polyacrylamide gel in reduced conditions and analyzed for TLR2 production by immunoblot using rabbit monoclonal antibody to TLR2 (Invitrogen, clone JM22-41, 1:1000) as detected by chemiluminescence for a 60 second exposure time.

### *L. monocytogenes* Trophoblast Invasion Measured by Gentamicin Survival Assay

Cells were cultured in 6-well plates and infected with 8 x 10^6^ WT *Lm*/well, centrifuged, and incubated 1 h at 37°C. Cells were then incubated for 30 min with gentamicin (15 µg/ml) and immediately washed and plated (1.5 h sample), or were pulsed with gentamicin for 30 min every 5 hours, for the time points 5, 10, 15, and 24 h. Cells were then washed three times in warm PBS and lysed by incubation with 0.2% Triton X-100 for 5 min at RT followed by plating of serial dilutions of the cell lysates on BHI agar plates for colony forming unit (CFU) determination. Apparent growth rate was calculated as ln (CFUt/CFUt_0_)/(t-t_0_) and doubling time = ln(2)/gr.

### Measurements of Bacterial Actin Tails and Clouds, and Bacterial Length

*For measuring the formation of F-actin clouds and comet tails* on cytosolic *L. monocytogenes*, PHT and HeLa cells, cultured in 24-well plates with glass coverslips, were infected with 10^6^ bacteria/well p*actA*-RFP *L. monocytogenes* for 1 h, followed by 30 min treatment with gentamicin, and 3.5 h incubation in cell culture medium without antibiotic. E-cadherin (only performed on trophoblasts) and F-actin fluorescence labeling were performed on fixed and TX-100 permeabilized cells. *For measuring the length of intracellular L. monocytogenes*, PHT were infected in duplicate in 24 well plate, with 10^5^ bacteria/well WT *L. monocytogenes* for 1 h, washed and incubated with gentamicin for 30 min. Cells were then continuously incubated with gentamicin for 24 h (no pulse), or cells were pulsed for 30 min with gentamicin every 5 hours. After 24 h, E-cadherin and bacteria were labeled as described above. Twenty sets of images were acquired per experimental condition. MetaMorph was used to measure the length in µm of RFP-fluorescent bacteria.

### RNA Extraction and RNAseq Using the Illumina Platform NovaSeq SP Flowcell

PHT and BeWo cells were infected, or not, in 6 well-cell culture plates with 4 x 10^6^ WT *Lm*/well for 1 h followed by 30 min gentamicin, and 3.5 h in gentamicin-free medium (5 h samples). Cells were pulsed with gentamicin for 30 min after 5 hours infection, and incubated in gentamicin free medium for another 5 hours (10 hours samples). Cells were lysed in TRIzol reagent (Invitrogen). PureLink RNA extraction kit with on-column DNase treatment (Invitrogen) was used to isolate RNA. Input RNA integrity number (RIN) were all > 7 and concentration was >100 ng/µl RNA, as determined with the Agilent 2100 Bioanalyzer (Agilent Technologies, Santa Clara, CA) and Qubit Fluorometer (Thermo Fisher). RNA-seq libraries were generated with NEBNext^®^ Ultra™ II Directional RNA Library Prep Kit for Illumina (NEB #E7760L) and NEBNext^®^ rRNA Depletion Kit with sample purification beads (NEB #E6350) with an input amount of 200 ng total RNA per sample. Libraries were pooled and sequenced on an Illumina NovaSeq SP flowcell in paired-end 150 bp format (Illumina, San Diego, CA) to a read yield between 70-80 million reads (equivalent to 35- 40 million clusters). Three independent experiments were performed to compare infected to non-infected, PHT and FSK-treated or not, BeWo cells.

### RNAseq Data Analyses

Raw fastq was aligned to human reference genome GRCh38 with hisat2 v2.1.0 (60, 61). Alignment QC was assessed by RSeQC. Gene wise counts were generated with featureCounts from the subread package v1.5.1 for genes annotated by Ensembl GRCh38.92 (62). Counts normalization and differential expression were performed with edgeR using a negative binomial distribution with a generalized linear model with a paired design (63). Genes were tested if at least half of the samples had an expression of 2 counts per million (CPM). Significant genes were defined as those with a false discovery rate (FDR) < 0.05 and absolute value (log2FC) > 1. The fold change of a gene was considered positive when its CPM was higher in *L. monocytogenes*-infected cells than in non-infected cells. Heatmaps were generated in R using package ComplexHeatmap. GO term and KEGG enrichment were performed with DAVID 6.8 for genes that were differentially expressed (64, 65). Pathway analysis was performed in Ingenuity Pathway Analysis.

### Quantitative Real-Time PCR

Cells were lysed in TRIzol and total RNA was isolated using PureLink RNA extraction kit with on-column DNase treatment (Invitrogen). Total RNA was quantified by spectrophotometry with a NanoDrop 2000 (Thermo Scientific) and 1-3 ug total RNA from each sample were reverse-transcribed into cDNA by ThermoScript RNase H^-^ reverse transcriptase (Invitrogen Life Sciences). Quantitative real-time PCR was performed using an equal amount of cDNA per sample on a Bio-Rad CFX Real-Time PCR Detection System, using primer sets specific to *IFNL1*, *IFNL2*, *IFNL3*, *TNF*, *IL1B*, *IL6*, *IL8*, and *IL10* with iQ SYBR Green Supermix. Primers were designed using an algorithm which we described earlier (66). Clustal Muscle 3.8 alignment for IFNL genes was used to verify specificity of primers. Primer’s efficiency was calculated using the following equation: E=10^(-1/slope)^. Gene expression was analyzed with the Bio-Rad CFX Maestro Software. Relative copy numbers (RCN) of selected genes of interest were normalized to the expression of two housekeeping genes (GAPDH and CAP-1) using the following equation: RCN = E^-ΔCt^ x 100, where E is the efficiency of PCR and ΔCt is the Ct_(target)_ – Ct_(reference)_ (reference=average of the two housekeeping genes) (66, 67).

### Cytokine Array and Lambda Interferon ELISA

Cell culture supernatants were collected from infected and control non-infected PHT, and FSK-treated or not BeWo cells. Supernatants were immediately centrifuged and frozen at -80°C. Thawed samples were pooled from three independent experiments and cytokine production was measured using the V-PLEX Human Biomarker 54-Plex Kit (Meso Scale Discovery). IFNL1, IFNL2, and IFNL3 ELISAs were performed using the human IL-29 (DY7246), IL-28A (DY1587), and IL-28B (DY5259) DuoSet ELISA kits (R&D Systems) per the manufacturer’s instructions.

### Colocalization of Cytokines (IL-8, IL-6, TNFα) With Fused and Non-Fused Trophoblasts

Primary mouse anti-IL-6 (R&D Systems, clone #1936, 1:33) and anti-IL-8 (R&D Systems, clone #6217, 1:33) antibodies were used in combination with secondary fluorescent antibodies to label infected PHT following PFA fixation and permeabilization by 0.3% TX-100. To distinguish fused from non-fused PHT, cells were colabelled using primary rabbit and βhCG (Abcam) and secondary fluorescent antibodies. Primary rabbit anti-TNFα (a gift from Dr. Mark Wewers, The Ohio State University, 1:100) (68) and mouse anti-E-cadherin antibodies were used with secondary fluorescent antibodies to label infected PHT following PFA fixation and permeabilization by 0.3% TX-100. Cytosolic *L. monocytogenes* expressed RFP and the dye DAPI was added post-fixation to visualize the host cell nuclei. The specificity of cytokine labeled was ensured by performing labeling with isotype control antibodies in parallel experiments, which led to low level background fluorescence. At least 30 sets of fluorescence images (RFP-*Lm*, DAPI, GFP-E-cadherin or -βhCG, far-red-cytokine) were acquired in each experimental condition (each image includes ∼ 300 host cell nuclei). Colocalization experiments between the cytokine and fusion marker (E-cadherin or βhCG), using the MetaMorph software, were performed on background corrected fluorescence images, and a threshold was applied to analyze the cytokine integrated fluorescence intensity relative to cell areas covered (colocalization), or not (segregation), by βhCG (highlights fused cells) or by E-cadherin (highlights non-fused cells). Data were expressed as average percentage colocalization ± SEM of one representative experiment (of 3 independent experiments with 3 placentas).

### Microscope Equipment

Images were acquired on a motorized, atmosphere-controlled inverted wide-field fluorescence microscope (Axio Observer D1, TempModule S1, Heating Unit XL S; Zeiss) equipped with a PZ-2000 XYZ series automated nano stage (Applied Scientific Instruments), 20X Plan Neofluar (numerical aperture [NA] = 0.5), 40X Plan Neofluar (NA = 1.3), and 100x Plan Apo (NA = 1.4) objective lenses, a high-speed Xenon fluorescence emission device (Lambda DG-4, 300 W; Sutter Instrument Company), a Lambda 10-3 optical emission filter wheel for fluorescence imaging, a SmartShutter to control the illumination for phase-contrast imaging (Sutter Instrument Company), a back-illuminated, frame-transfer electron-multiplying charge-coupled device (EMCCD) camera (Cascade II:512; Photometrics), and an ORCA-FLASH 4.0 V2 digital complementary metal-oxide –semiconductor camera (Hamamatsu). The filter sets for fluorescence were purchased from Chroma Technology Corporation and were as follows: DAPI (49000), Alexa Fluor 488 (49002), Alexa Fluor 568 (49005), and Cy5 (49006). Images were acquired and analyzed using MetaMorph imaging software (Molecular Devices).

### Statistical Methods

All experimental work involved at least three independent experiments, each performed in duplicate. All statistical analyses were performed using GraphPad Prism 7 and 8. Secretion of hCG, bacterial association, bacterial internalization, and fusion percentages were presented as mean ± SEM. Statistical significance of association, internalization, and fusion was determined by linear mixed effects models and p-values were adjusted with Holm-Sidak’s procedure, unless otherwise stated. For ELISA analysis, data were first normalized to internal controls or the loading standard to reduce variation before analysis. Statistical methods are indicated in figure legends, n indicates the number of independent experiments (each involving a different placenta).

## Results

### InlA- and ActA-Mediated *L. monocytogenes* Uptake Are Ineffective in Syncytia

**Figure 1:**
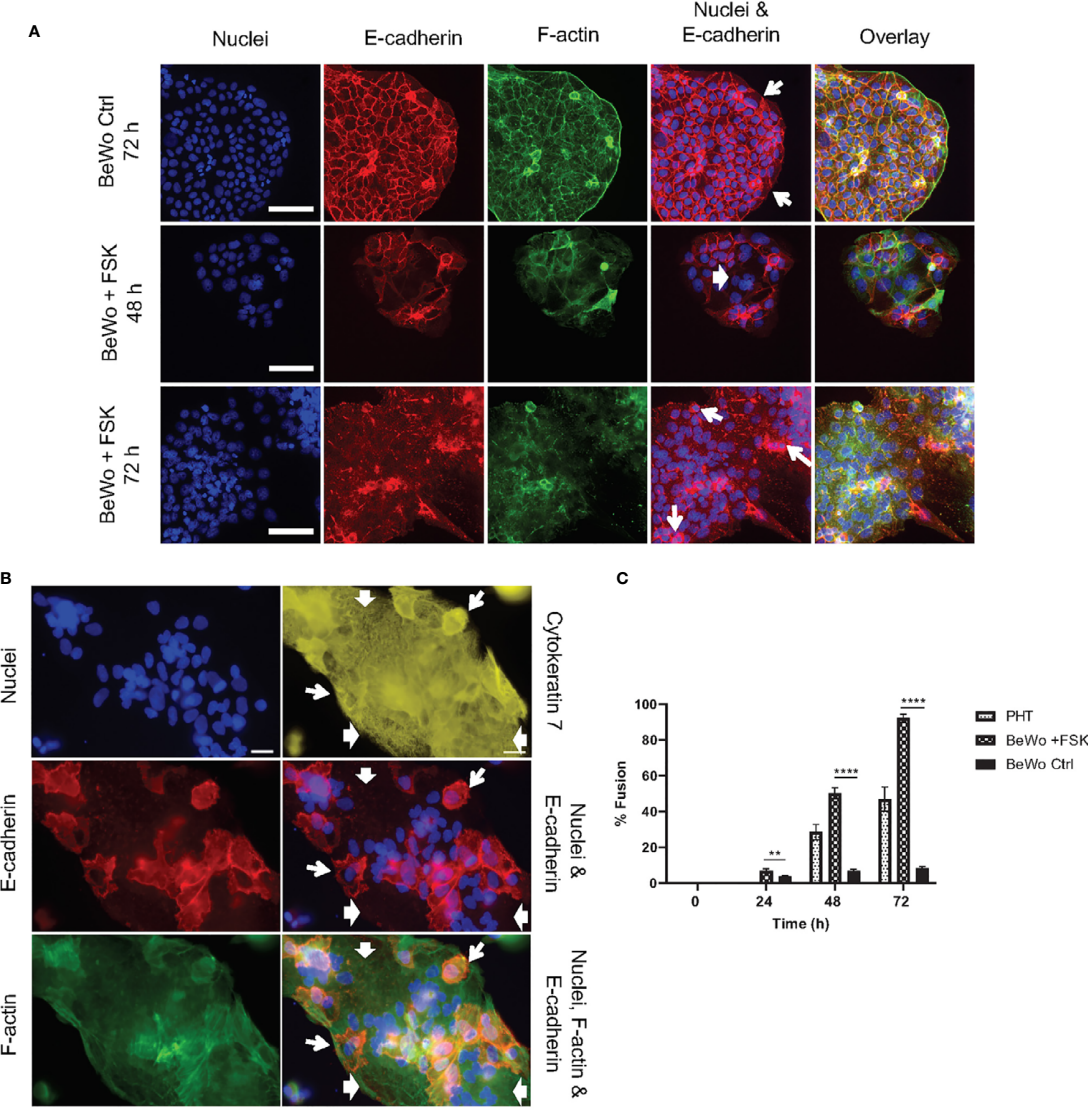
Trophoblast culture models. **(A)** BeWo cells were cultured 24 h prior to daily treatment with 20 µM forskolin (BeWo +FSK), or with 0.1% control DMSO (BeWo Ctrl), for up to 72 h. Cells were fixed and fluorescently labeled to visualize nuclei (DAPI), F-actin (phalloidin-Alexa Fluor 568), and intercellular junctions (E-cadherin, Alexa Fluor 488-conjugated antibodies). Fluorescence images were acquired with a 20X objective (scale bar = 100 μm). **(B)** Primary human trophoblasts (PHT) were cultured for 72 h before fixation and fluorescence labeling to visualize nuclei (DAPI), intercellular junctions (E-cadherin, Alexa Fluor 568-conjugated antibodies), F-actin (phalloidin-AlexaFluor 488), and trophoblasts (cytokeratin 7, Alexa Fluor 647-conjugated antibodies). Fluorescence images were acquired with a 40X objective (scale bar = 50 μm). In **(A, B)**, wide arrows highlight examples of fused cells visualized by the absence of E-cadherin and the presence of multiple nuclei. Narrow arrows highlight some mononuclear non-fused cells delineated by continuous E-cadherin junctions. **(C)** PHT were analyzed after 48 h and 72 h of culture and BeWo cells were analyzed 0, 24, 48, and 72 h after FSK treatment. At least 2,000 – 3,000 nuclei were counted per experimental condition. Data are expressed as Percent Cell Fusion ± SEM. Statistical significance was determined using the Two-stage linear step-up procedure of Benjamini, Krieger and Yekutieli, with Q = 1%. Each row was analyzed individually, without assuming a consistent SD. **P = 0.0083, ****P < 0.000001 (n = 4 placentas for PHT, n = 3 for BeWo).

**Figure 2:**
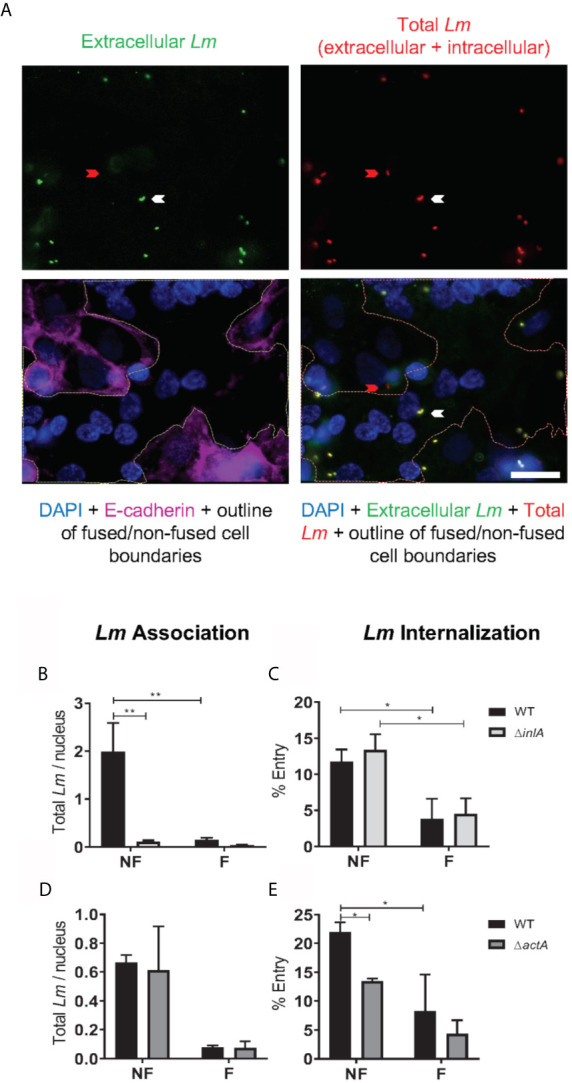
*L. monocytogenes* association and internalization into primary human trophoblasts. PHT were infected with isogenic *L. monocytogenes* (*Lm*) WT, Δ*inlA*, or Δ*actA* at MOI 1 **(A–C)** or 0.5 **(D, E)** for 1 h at 37°C. Cells were washed, fixed and labeled to enumerate bacterial association and internalization into fused (F) and non-fused (NF) trophoblasts by fluorescence microscopy. Ten fields of phase-contrast and fluorescence images (DAPI, total *Lm*-Alexa Fluor 488, extracellular *Lm*-Alexa Fluor 568, and E-cadherin-Alexa Fluor 647) were randomly acquired from duplicate slides (20 image sets), for each experimental condition using a 100X objective. **(A)** Representative images. The red arrow points towards an intracellular *Lm* within a non-fused trophoblast. The white arrow points towards an extracellular *Lm* on a fused cell. A white outline was drawn on the overlay images to delineate the boundaries between non-fused and fused trophoblasts. Scale bar = 20 µm. **(B–E)** The numbers of total *Lm* (Nt), extracellular *Lm* (Ne), and DAPI-labeled host cell nuclei (Nn) were counted in fused and non-fused PHT. *Lm* Association **(B, D)** was calculated as Nt/Nn. *Lm* Internalization **(C, E)** was calculated as [(Nt – Ne)/Nt] x 100. Data are expressed as the average ± SEM (**B, C** n = 5) of 3 independent experiments (3 placentas) (**D, E** n = 3). Statistical significance was determined by 2-way ANOVA. P-values were adjusted using Holm-Sidak’s multiple comparisons test, **P = 0.0024, *P = 0.0266 **(B, C)**; *P = 0.0304, 0.0486 **(E)**. All cell counts corresponding to data analysis of **Figure 2** are summarized in **Figure S9**.

Two cell culture models were used to establish the mechanisms that confer syncytia enhanced resistance to *L. monocytogenes* infection in comparison to non-fused trophoblasts: (1) primary human mononuclear trophoblasts obtained from term placentas (PHT), which spontaneously form syncytia in culture, and (2) the BeWo cell line that forms syncytia upon forskolin (FSK) treatment (**Figure 1**) (52, 69). Syncytia formation was measured as the percentage of fused cells defined as cells with three or more nuclei, based on visualization of intercellular junctions (E-cadherin labeling) and number of nuclei per cell (DAPI labeling) (**Figures 1A, B**). PHT cultured for 72 h and BeWo cells treated with FSK for 48 h reached a similar percentage of fusion (~50%)(**Figure 1C**); therefore, these cell culture conditions were used in all experiments presented in this article. BeWo cells (FSK-treated or not) and PHT were infected for 1 h with isogenic wild-type (WT), Δ*inlA*, Δ*inlB*, Δ*hly*, and Δ*actA L. monocytogenes*. After chemical fixation and fluorescent labeling of bacteria and trophoblasts, the efficiencies of *L. monocytogenes* association and internalization into syncytia versus non-fused trophoblasts were measured by fluorescence microscopy (**Figure 2A**). Data showed that syncytia are significantly less susceptible (~20-fold) to *L. monocytogenes* infection than non-fused trophoblasts, as previously reported (**Figures 2B–E**) (70). As expected, this drastic decrease in syncytia invasion is primarily due to the significant decrease in InlA/E-cadherin-mediated *L. monocytogenes* invasion (8). We then dissected if the InlA/E-cadherin interaction mediates bacterial attachment and/or internalization. Not surprisingly, we found that bacterial association to non-fused PHT, which express high levels of E-cadherin, is InlA-dependent; whereas, WT and Δ*inlA* similarly display low association to syncytia, which express faint E-cadherin levels (**Figures 1B**, **2B**). However, InlA/E-cadherin interaction did not play a role in bacterial internalization into PHT (**Figure 2C**). We show that *L. monocytogenes* internalization efficiency is significantly decreased in syncytia in comparison to non-fused cells (**Figures 2C, E**). We identified a significant role for ActA in bacterial internalization into non-fused PHT, but this role was no longer significant in syncytia (**Figure 2E**). Using the BeWo cell model, we observed that InlA controlled both *L. monocytogenes* association and internalization, whereas no role for ActA was observed in this cell line (**Figure S2**). Also, no role for LLO or InlB in *L. monocytogenes* association and internalization was observed in PHT or BeWo cells, as we and others previously reported with this bacterial strain (data not shown) (36, 71). In conclusion, our studies support that both InlA and ActA play a significant role in promoting *L. monocytogenes* uptake by mononuclear trophoblasts, but this role is lost in the SYN. The cell line BeWo does not constitute a model for the study of the role of ActA in trophoblast invasion, which highlights the importance of working with primary cells.

### *L. monocytogenes* Cytosolic Doubling Time Is Similar in Syncytia and Non-Fused Trophoblasts

**Figure 3:**
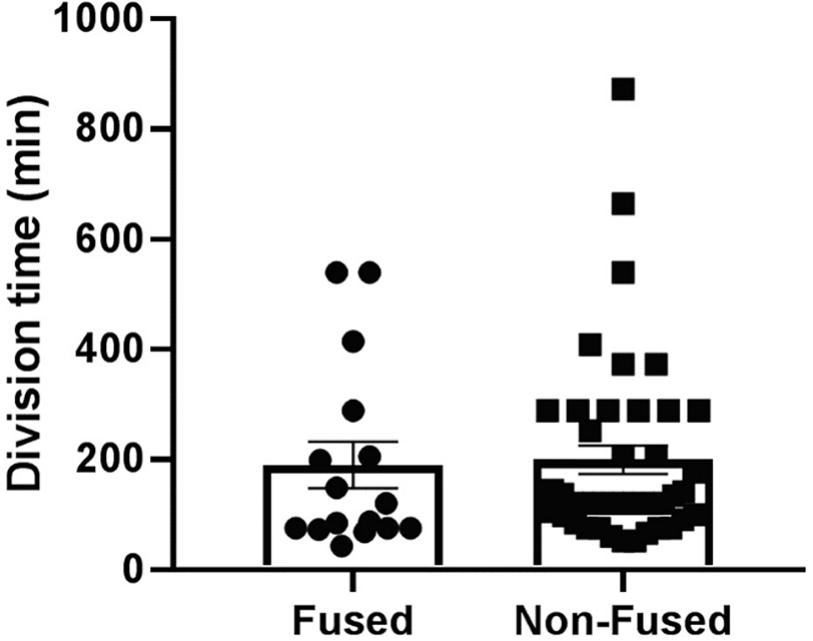
*L. monocytogenes* cytosolic doubling time. PHT were plated in 35 mm diameter imaging dishes at 2x10^6^ cells/dish and cultured for 72 h. Cells were infected with pactA-RFP *L. monocytogenes* at MOI 1 for 5 h. Cells were transferred to the temperature-controlled microscope stage. The plasma membrane was labeled with CellMask Deep Red dye and the nuclei with Hoechst 33342 (**Figure S3**). At least four fields of view of infected fused and infected non-fused PHT were selected and memorized. Initial sets of phase-contrast and fluorescence (RFP, DAPI, Deep Red) images were acquired immediately and at 1 h time intervals at the four positions for 2 hours. Z-stacks of the fluorescent bacteria were acquired to facilitate bacterial counting in 3D. Division time was calculated based on the difference between the initial and final DSRED images using the following equations: growth rate (gr) = (ln [N(t)/N(0)])/t and doubling time = ln (2)/gr, where N(t) is the final number of bacteria, N(0) is the initial number of bacteria, and t is the time point in hours. (n = 3 placentas).

To evaluate the *L. monocytogenes* intracellular doubling time, a gentamicin survival assay was performed in PHT and BeWo cells infected for up to 24 h (**Table 1**). The bacterial load (CFUs) reached a plateau as early as 10 h post-infection, which reflects that cells were over-infected and/or became permeable to the antibiotic gentamicin used to kill extracellular bacteria. Comparison of CFUs between 1.5 h and 5 h infection led to the calculation of an apparent intracellular doubling time: 70.8 min ± 5.0 (SEM) in PHT, 69.0 min ± 5.5 (SEM) in FSK-treated BeWo, and 64.3 min ± 3.8 (SEM) in control non-fused BeWo cells. Because this method could not distinguish syncytia from non-fused cells in PHT, we also measured the bacterial doubling time by live-cell fluorescence microscopy. PHT were infected with p*actA*-RFP *L. monocytogenes*, which expresses the red fluorescent protein under the control of a promoter that is only active when bacteria have reached the cytosol (11). In these experiments, after 5 h of infection in the absence of gentamicin, cells were transferred to the atmosphere-controlled microscope, and the fluorescent dyes CellMask Deep Red and Hoechst 33342, were added to label the host cell plasma membrane and nuclei, respectively, thereby allowing us to distinguish fused from non-fused cells (**Figure S3**). We found, by counting the number of RFP-positive bacteria between 5 h and 7 h of infection, that the average doubling times of *L. monocytogenes* were similar in fused [190.0 min ± 39.4 (SEM)] and non-fused PHT [199.4 min ± 29.6 (SEM)](**Figure 3**). Surprisingly, we observed by fluorescence microscopy that a proportion of intracellular *L. monocytogenes* were elongated in both fused and non-fused PHT after 10 h of infection, which may be a manifestation of bacterial stress (72). This was not observed in extracellular bacteria or in BeWo cells, regardless of their fusion status and infection time, for up to 24 h (data not shown). To determine the potential contribution of gentamicin, we measured the length of intracellular *L. monocytogenes* in PHT infected for 24 h, either continuously exposed to gentamicin or subjected to 30 min gentamicin pulses every 5 h (**Figure S4**). We found that the proportion of elongated bacteria was lower when cells were pulsed with gentamicin, reflecting that penetration of the antibiotic into the PHT could account for bacterial elongation, at least partially. In conclusion, *L. monocytogenes* intracellular doubling time is similar in the SYN and mononuclear trophoblasts (in PHT and BeWo) at least for the first 7 hours of infection. Interestingly, we observed that the division time significantly increases after 5 h of infection independently of the cell fusion status. In parallel, we observed bacterial elongation after 10 h of infection, but this phenotype was influenced, at least partially, by the presence of gentamicin in the cell culture medium.

**Table 1:** Gentamicin survival assay.

Time (h)	PHT	BeWo (+FSK)	BeWo (control)
1.5	4.3E6 ± 2.2E6	1.4E6 ± 7.2E5	2.7E6 ± 9.8E5
5	3.6E7 ± 1.6E7	1.1E7 ± 3.8E6	2.5E7 ± 4.7E6
10	2.5E7 ± 8.3E6	3.3E7 ± 3.9E6	2.8E7 ± 5.8E6
15	3.5E7 ± 1.6E7	2.5E7 ± 2.6E6	7.6E6 ± 7.1E6
24	1.1E7 ± 3.8E6	8.4E6 ± 2.9E6	1.4E6 ± 2.3E5

PHT and BeWo cells were infected with WT L. monocytogenes (MOI 1) for 1 h at 37°C followed by a 30 min incubation with gentamicin. Cells were washed and incubated for a total of 1.5, 5, 10, 15, and 24 h including 30 min gentamicin pulses performed every 5 h. Data are presented as the average CFUs/ml ± SEM (n = 3). Based on CFUs between 1.5 and 5 h, apparent doubling time from 1.5 to 5 h for PHT was 70.8 ± 5.0 min, for Fused BeWo 69.0 ± 5.5 min, and for Non-Fused (control) BeWo 64.3 ± 3.8 min.

### The Proportion of Motile *L. monocytogenes* and the Average Bacterial Speeds Are Similar in Syncytia and Non-Fused Trophoblasts

**Figure 4:**
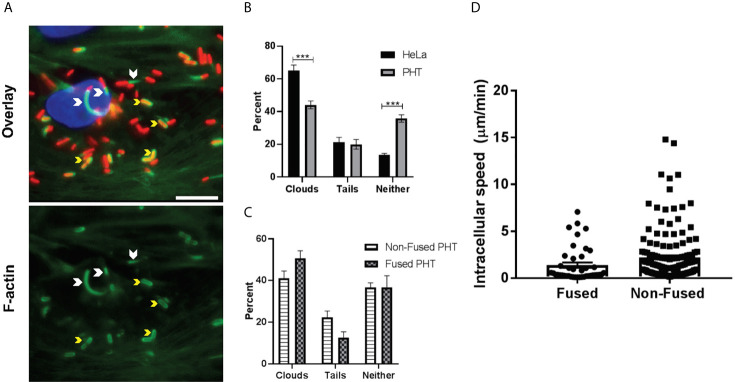
Measure of F-actin clouds and comet tails. PHT and HeLa cells were infected with p*actA*-RFP *L. monocytogenes* for a total of 5 h (MOI 1). Cells were fixed and labeled with DAPI, phalloidin-Alexa Fluor 488, and anti-E-cadherin antibodies conjugated to Alexa Fluor 647 (PHT only). Fluorescence images were acquired with the 100X objective and analyzed for the presence of F-actin comet tails and clouds. **(A)** Representative images, arrowheads point out F-actin tails and yellow arrows depict F-actin clouds. Scale bar = 50 µm. **(B, C)** At least 190 bacteria were counted per experimental condition in each experiment. Data are presented as Average percentages ± SEM (n = 3 (3 placentas), each experiment performed in duplicate). Statistical significance was determined by two-way ANOVA, Holm-Sidak’s procedure was used for multiple comparison adjustment. ***P = 0.0003, P = 0.0002 (left to right). **(D)** Intracellular L. monocytogenes speed was determined by acquiring Z-stacks with the 100x objective every 5 sec for 5 min to measure bacterial motility. Bacteria were tracked through each plane of the Z-stack using the multi-line function of Metamorph. Each plane is considered 5 sec and the total µm/min was calculated based on the total length of each bacterial track through the Z-stack.

ActA-mediated *L. monocytogenes* intracellular motility, *via* the assembly of the host actin cytoskeleton at one bacterial pole, promotes *L. monocytogenes* spreading from cell-to-cell (45). Because PHT syncytialization is paralleled with a decrease in actin stress fibers and an increase in the G-/F-actin ratio, we hypothesized that *L. monocytogenes* motility could be altered in syncytia (15, 69). We thus measured if *L. monocytogenes* could nucleate host actin as efficiently in syncytialized and non-fused PHT. As a control, we used HeLa cells in which *L. monocytogenes* motility has been extensively studied (73–75). PHT and HeLa cells were infected with p*actA*-RFP *L. monocytogenes*. In these assays, cells were treated with gentamicin for 30 min following infection and were then cultured in gentamicin-free medium for the remaining 3.5 h. After fixation and labeling, the percentage of cytosolic bacteria that could recruit actin clouds or comet tails was enumerated by fluorescence microscopy (**Figure 4A**). About 20% of RFP-expressing bacteria formed comet tails in PHT, similar to HeLa cells (**Figure 4B**). However, there was a significant difference in the percentages of bacteria decorated with actin clouds (about 44% in PHT and 65% in HeLa) and bacteria devoid of an actin coat (about 36% in PHT and 14% in HeLa). In syncytia, the percentage of actin tails was lower and the percentage of clouds higher in comparison to non-fused PHT, but these differences did not reach statistical significance (**Figure 4C**). As a more accurate method to assess bacterial motility, we measured cytosolic speed of RFP-expressing *L. monocytogenes* by live-cell fluorescence imaging 5 h post-infection. We found that the percentage of non-motile versus motile bacteria was 76.2% ± 2.9 vs 23.8% ± 2.9, respectively in non-fused and 74% ± 15.4 vs. 26% ± 15.4 in syncytia (n=4 placentas), confirming data obtained from fixed cells. The average speed of motile bacteria was also similar in syncytia and non-fused PHT (**Figure 4D**). Collectively, our data support that the barrier function of the SYN operates *via* blocking *L. monocytogenes* entry into the SYN, but not bacterial intracellular proliferation or motility, at least during the first 7 h of infection.

### Transcriptome of Primary Trophoblasts Infected by *L. monocytogenes* Reveals a Pro-Inflammatory Switch With Upregulated Signature Genes Associated With Pregnancy Complications

**Figure 5:**
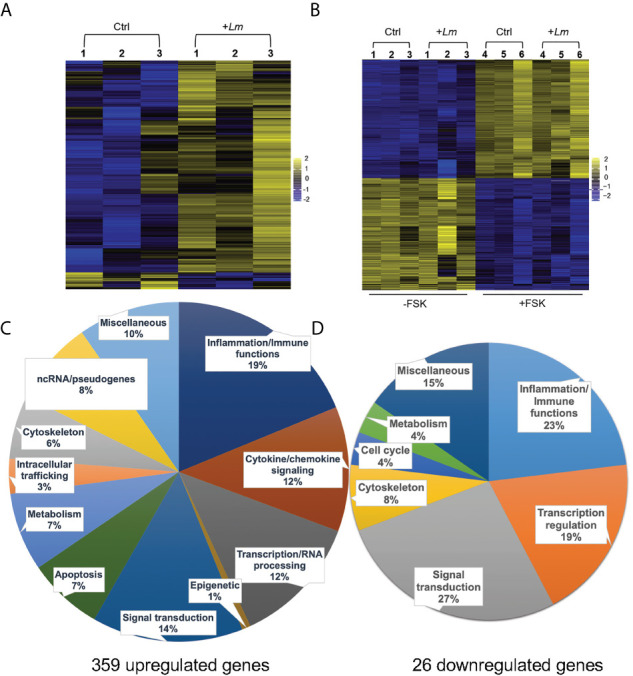
RNAseq analysis of *L. monocytogenes* infected trophoblasts. Heatmaps of PHT **(A)**, and FSK-treated (+FSK) or DMSO-treated control (-FSK) BeWo cells **(B)**, infected for 5 h (+*Lm*) or control non-infected (Ctrl). RNA was extracted from infected and control cells for RNAseq analysis. RNA-seq counts were generated by featureCounts from subread-1.5.1 for genes annotated by GRCh38.92. Count normalization and differential expression was performed using edgeR. Heatmaps were generated by plotting genes with log_2_(FC) > 1 and < -1 and p value < 0.05. Scale is based on log_2_(CPM) from -2 to +2 (n = 3 placentas, and 3 independent experiments involving BeWo cells). Genes were clustered based on similarity of expression profiles. **(C, D)** The upregulated and downregulated DEG were classified based on their known function.

We performed RNAseq analysis to determine how *L. monocytogenes* infection for 5 h affects the trophoblast transcriptome. Three independent experiments involving 3 different placentas were performed comparing infected to non-infected PHT. In parallel, we analyzed infected and non-infected FSK-treated and control BeWo cells (**Figures 5A, B**). We found that among the 12,275 detected PHT genes, corresponding to genes which expression was at least 2 counts per million (CPM) in half of the samples, a total of 385 genes were differentially expressed (DEG) upon infection (**Figures 5C, D**). These include 359 upregulated genes, whereas only 26 genes were downregulated. DEGs were defined as genes with log_2_ (FC) >1 or <-1, and FDR-adjusted P < 0.05 comparing non-infected and infected cells at time point 5 h. In contrast, FSK treatment, but not infection, significantly affected the BeWo cell transcriptome (**Figure 5B**). Importantly, RNAseq data were validated by real-time quantitative polymerase chain reaction (RT-qPCR) analysis of a collection of DE cytokine genes using 5 h infected versus non-infected trophoblasts (PHT isolated from 3 additional placentas and independent BeWo cell samples) (**Tables S1**, **S2**). As a first analysis of the transcriptomic changes, PHT DEG were grouped into functional categories (**Figures 5C, D**). The largest category of genes controls inflammation and immune functions (31% of total upregulated and 23% of total downregulated genes). The prevalence of immune genes was also illustrated by the top 50 DE protein-coding genes, defined as the highest absolute log_2_FC values, (**Table 2**), 76% of which have inflammatory and immune functions, with 60% of genes encoding cytokines, chemokines, their receptors and regulators. The second largest category includes genes controlling cell signaling (14% of upregulated and 27% of downregulated DEGs). The third category includes genes encoding transcription factors and co-activators, and enzymes controlling the epigenome (13% of upregulated and 19% of downregulated DEGs). We found that 41 upregulated DE genes encode transcriptional regulators (**Table S3**). Additional upregulated DEGs are involved in the regulation of apoptosis, metabolism, and the cytoskeleton. Of note, genes involved in cell signaling, transcription, and other categories cited above, include genes regulating immune responses, thereby leading to an underestimation of the overall proportion of genes involved in immune functions.

**Table 2:** Top 50 Induced Genes.

Ensembl Gene ID	gene_name	logTPM	logCPM	logFC	FDR	Ensembl Gene ID	gene_name	logTPM	logCPM	logFC	FDR
ENSG00000164400	CSF2	5.2	2.6	7.8	2.7E-47	ENSG00000125657	TNFSF9	3.4	1.9	4.4	1.6E-19
ENSG00000115009	CCL20	5.2	3.3	7.7	1.6E-67	ENSG00000124882	EREG	6.6	6.7	4.4	6.6E-35
ENSG00000275302	CCL4	6.0	4.7	7.4	8.6E-113	ENSG00000158050	DUSP2	5.2	4.0	4.3	1.3E-35
ENSG00000108342	CSF3	3.8	2.6	7.1	9.2E-35	ENSG00000143479	DYRK3	1.8	1.5	4.2	5.6E-16
ENSG00000117090	SLAMF1	2.6	2.2	7.1	4.2E-48	ENSG00000169429	CXCL8	10.2	9.1	4.2	3.8E-19
ENSG00000277632	CCL3	8.4	6.7	6.7	1.8E-72	ENSG00000134460	IL2RA	3.5	2.9	4.0	5.2E-30
ENSG00000050730	TNIP3	3.0	2.3	6.6	2.5E-36	ENSG00000090339	ICAM1	7.6	7.2	4.0	5.0E-42
ENSG00000125538	IL1B	8.3	7.6	6.5	3.0E-64	ENSG00000170209	ANKK1	4.7	3.7	4.0	1.4E-31
ENSG00000276085	CCL3L1	6.5	4.8	6.2	3.9E-54	ENSG00000023445	BIRC3	4.8	5.2	4.0	9.5E-56
ENSG00000123689	G0S2	6.6	4.1	6.2	4.3E-83	ENSG00000171174	RBKS	4.0	3.6	3.8	1.1E-37
ENSG00000110944	IL23A	6.1	4.0	6.0	9.9E-75	ENSG00000184371	CSF1	5.3	5.3	3.8	3.5E-38
ENSG00000276070	CCL4L2	5.2	3.4	5.9	1.4E-57	ENSG00000160326	SLC2A6	4.5	4.1	3.8	1.8E-42
ENSG00000126353	CCR7	6.4	5.4	5.9	4.5E-92	ENSG00000140379	BCL2A1	5.2	3.0	3.8	1.3E-26
ENSG00000197110	IFNL3	4.4	1.6	5.6	2.3E-17	ENSG00000172548	NIPAL4	3.2	3.2	3.7	8.2E-26
ENSG00000182393	IFNL1	3.9	1.3	5.5	2.3E-22	ENSG00000081041	CXCL2	7.5	5.9	3.7	1.8E-31
ENSG00000183709	IFNL2	4.8	1.8	5.5	5.2E-15	ENSG00000073756	PTGS2	6.2	6.3	3.5	1.7E-22
ENSG00000049249	TNFRSF9	3.3	3.5	5.3	1.5E-56	ENSG00000136244	IL6	6.1	5.0	3.5	5.8E-28
ENSG00000241794	SPRR2A	4.8	1.8	5.3	1.5E-23	ENSG00000137265	IRF4	1.3	1.5	3.4	1.8E-12
ENSG00000056558	TRAF1	5.4	5.8	5.1	9.9E-75	ENSG00000115008	IL1A	6.9	5.6	3.4	7.5E-15
ENSG00000123610	TNFAIP6	4.6	2.9	4.9	4.7E-43	ENSG00000109321	AREG	4.7	3.1	3.3	1.8E-23
ENSG00000232810	TNF	6.9	5.4	4.8	1.7E-45	ENSG00000026751	SLAMF7	4.7	4.6	3.2	1.3E-30
ENSG00000136634	IL10	3.1	1.8	4.7	3.8E-11	ENSG00000163735	CXCL5	2.3	1.2	3.1	1.1E-06
ENSG00000163734	CXCL3	7.1	5.9	4.6	9.3E-57	ENSG00000183484	GPR132	4.4	4.4	3.1	1.5E-35
ENSG00000172602	RND1	3.3	2.5	4.5	1.8E-30	ENSG00000135373	EHF	2.9	2.6	3.0	2.2E-11
ENSG00000163739	CXCL1	6.8	4.8	4.4	1.5E-49	ENSG00000136689	IL1RN	6.7	6.2	3.0	9.9E-22

The top 50 upregulated PHT DEG are represented based on log_2_(FC) and FDR. Data are the average of three independent experiments. TPM, transcripts per million; CPM, counts per million; FDR, false discovery rate.

We then performed gene enrichment and functional annotation analysis to gain understanding of specific pathways affected in PHT upon *L. monocytogenes* infection. We used the DAVID Bioinformatics Resources (6.8, NIAID/NIH DAVID) to analyze the 330 upregulated protein-coding genes. The top biological processes (BP) were identified using P value and FDR<0.05, EASE value of 0.05 (**Table 3**). The BP were dominated by immune responses, inflammation, positive regulation of transcription, signal transduction, and negative regulation of apoptosis, which are in accordance with the gene functional categories reported in **Figure 5C**. The molecular functions also highlighted transcriptional regulation and cytokine-based signaling (**Table 3**). The Kyoto Encyclopedia of Genes and Genomes (KEGG) pathway analysis (P value and FDR<0.05, ease value 0.05) allowed us to identify the most significant immune signaling pathways (**Table 3**). Consistent with the detection of Gram-positive bacteria by pattern recognition receptors, these pathways include toll-like receptor and NOD-like signaling. The most significant pathways included bacterial detection *via* TLR2 leading to the transcriptional activation of genes such as CXCL8, CCL3, and CCL4 (among others) that encode potent neutrophil and monocyte chemokines, and genes encoding the pro-inflammatory cytokines TNFα, IL-6, and IL-1β (**Figure S5A**). This finding is in accordance with the expression of TLR2 by fused and non-fused PHT and on the SYN and trophoblasts of human placentas, as previously demonstrated by others (**Figures S5B**, **C**) (76). Autocrine stimulation by cytokines is expected to amplify the PHT inflammatory responses, with TNFα as a prevailing pathway (**Figure S6**). Also consistent with TLR2- and TNFα- pathways, MAPK, PI3K-Akt, JAK-STAT, and NF-κB genes were upregulated (**Table 3** and **Figures S5**, **S6**). Gene enrichment analysis *via* Genetic Association Database (GAD) revealed sets of signature genes associated with infectious diseases (syncytial virus, HIV), cancer (multiple myeloma, Hodgkin disease and ovarian cancer), and rheumatoid arthritis, which were predominantly pro-inflammatory genes. Most relevant to listeriosis in pregnancy, 32 inflammatory genes (including 13 genes present in the top 50 DEGs) were associated with chorioamnionitis, placental infection and premature birth (**Table 3** and **Table S4**). In conclusion, RNAseq analysis of *L. monocytogenes*-infected human trophoblasts indicated the bacterial sensing pathways and resulting pro-inflammatory transcriptional circuitry. Importantly, this analysis also identified in *L. monocytogenes* infected PHT signature genes associated with poor pregnancy outcomes.

**Table 3:** Gene Enrichment and Annotation.

Biological Process	# of genes	P-value	FDR
Inflammatory response (GO: 0006954)	56	4.00E-34	8.40E-31
Immune response (GO: 0006955)	45	1.90E-21	2.00E-18
Positive regulation of transcription from RNAP II promoter (GO: 0045944)	49	2.20E-10	5.00E-08
Cell-cell signaling (GO: 0007267)	23	1.20E-09	2.00E-07
Signal transduction (GO: 0007165)	51	6.70E-09	1.00E-06
Positive regulation of gene expression (GO: 0010628)	21	6.00E-08	7.00E-06
Apoptotic process (GO: 0006915)	30	4.40E-07	4.10E-05
Innate immune response (GO: 0045087)	25	1.00E-06	7.80E-05
Negative regulation of apoptotic process (GO: 0043066)	25	2.70E-06	1.80E-04
Negative regulation of cell proliferation (GO: 0008285)	23	3.10E-06	2.00E-04
**Molecular Function**	**# of genes**	**P-value**	**FDR**
Cytokine activity (GO: 0005125)	24	9.10E-14	4.50E-11
Protein binding (GO: 0005515)	191	7.80E-05	9.50E-03
Transcription factor activity, sequence-specific DNA binding (GO: 0003700)	30	4.20E-03	1.60E-01
Identical protein binding (GO: 0042802)	23	1.60E-02	3.40E-02
**KEGG Pathways Induced**	**# of genes**	**P-value**	**FDR**
Cytokine-cytokine receptor interaction	41	1.00E-22	1.50E-20
TNF signaling pathway	27	2.90E-19	2.00E-17
NF-kappa B signaling pathway	20	2.20E-13	7.50E-12
Toll-like receptor signaling pathway	20	9.20E-12	2.10E-10
NOD-like receptor signaling pathway	15	5.10E-11	8.90E-10
Jak-STAT signaling pathway	18	9.70E-08	7.50E-07
Cytosolic DNA-sensing pathway	13	4.00E-08	3.30E-07
Chemokine signaling pathway	21	2.90E-08	2.70E-07
MAPK signaling pathway	19	5.50E-05	2.90E-04
**Disease Ontology**	**# of genes**	**P-value**	**FDR**
Chorioamnionitis|Fetal Membranes, Premature Rupture|Infection of amniotic sac and membranes	27	1.10E-14	2.60E-12
Inflammation|Premature Birth	21	1.70E-12	2.30E-10
Premature Birth	17	1.60E-10	1.30E-08

Analysis of the 330 upregulated DE protein-coding genes was performed with DAVID Bioinformatics Resources functional annotation. Significantly enriched activities from GO aspects for biological processes, molecular functions, KEGG pathways, and GAD for disease ontology. FDR are represented based on a -log_10_ scale (P value and FDR < 0.05 and EASE value of 0.05), the number of genes for each aspect is indicated as well as the corresponding FDR value, and the GO terms are between parentheses. DAVID, Database for annotation, visualization, andintegrated discovery; GO, gene ontology; KEGG, Kyoto Encyclopedia of Genes and Genomes; GAD, Genetic Association Disease Database; FDR, false discovery rate; EASE, expression analysis systematic explorer.

### The Secretome of *L. monocytogenes*-Infected Trophoblasts Confirms the Transcriptional Pro-Inflammatory Reprogramming and Revealed the Production of Tolerogenic Cytokines

In parallel to the preparation of PHT samples for RNAseq, cell culture supernatants were collected from infected and non-infected trophoblasts to measure the production of 54 inflammatory mediators. Cell culture supernatants were collected from 3 independent experiments and were pooled at the time of the assay. We used a V-PLEX Human Biomarker 54-Plex that includes 54 inflammatory mediators and growth factors that play key roles in inflammation and immunity. This assay was selected for its high dynamic range and sensitivity. Because primary trophoblasts have been shown to produce IFNλ and because *L. monocytogenes* was shown to induce the production of IFNλ by epithelial cell lines, we also used ELISA kits to measure IFNλ1, IFNλ2, and IFNλ3 (77, 78). *L. monocytogenes* infection for up to 10 h increased by at least 2-fold (average log_2_(FC)>1) the release of 12 mediators including chemokines (CXCL8, CCL3, CCL4, CCL20, CCL2, and CXCL10), pro-inflammatory cytokines (TNFα, IL-6, IL-1α, IL-1β), and anti-inflammatory cytokines (IL-10 and IL-1RA) (**Table 4**). Four immune mediators were produced in similar amounts by infected and non-infected PHTs (IL-27, VEGFR1, IFNλ3, and FGF2). Importantly, the fold-change of these mediators at the mRNA and protein levels were very similar 5 h post-infection, which supports that the increased production of cytokines and chemokines is most generally regulated at the transcriptional level (**Table 4**). Importantly, these findings also support the pro-inflammatory pathways identified by RNAseq, including the production of cytokines and chemokines. The transcripts encoding IFNλs were upregulated upon *L. monocytogenes* infection, as previously reported (78). IFNλ3 was constitutively produced by PHT, but its concentration remained unaffected by infection in our experimental conditions (**Table 4**). To rule out cell dysfunction or a failure to detect IFNλs, we infected PHTs with the Sendai virus from 5 h to 24 h, leading to the conclusion that viral infection significantly increased both transcripts and cytokine release (**Table S5**) (78). Overall, the PHT secretome is congruent with the transcriptomic data (with the exception of IFNλs) and supports the notion that trophoblasts play a role in the detection of *L. monocytogenes* and respond to this bacterium by producing pro-inflammatory mediators including cytokines and chemokines. Of importance, cytokines that play key roles in fetal tolerance, such as IL-27, IL-10, and IL-1RA, were also produced by infected trophoblasts, either at similar or increased levels compared to non-infected cells (**Table 4**) (79–81). The production of cytokines associated with pregnancy complications (premature birth and infections of the placental/fetal unit, **Table S4**) was also increased in *L. monocytogenes*-infected trophoblasts (CCL2, CCL3, IL-8, CSF2, IL-1α/β, IL-1RA, IL-10, GM-CSF, IL-6, and TNFα). This latter finding supports a potential role for trophoblasts in the generation of deleterious inflammation leading to poor pregnancy outcomes.

**Table 4:** PHT cytokine array.

	5 h			10 h			5 h RNAseq Log_2_(FC)
Cytokine	NI	*+Lm*	Log_2_(FC)	NI	*+Lm*	Log_2_(FC)	
**MIP-1α (*CCL3*)**	<2.83*	832	≥8.20	<2.83*	1360	≥8.91	6.69
**MIP-1β (*CCL4*)**	3.17	516	7.35	0.34	444	10.34	7.42
**IL-8 (*CXCL8*)**	50	3860	6.27	135.60	12324	6.51	4.17
**MIP-3α (*CCL20*)**	1.36	44	5.01	3.30	76	4.52	7.72
**MCP-1 (*CCL2*)**	2.04	14.24	2.81	2.28	10.52	2.21	2.38
**MCP-4 (*CCL13*)**	7.56	23.92	1.66	<2.66*	30.16	≥3.50	–
**IP-10 (*CXCL10*)**	18.36	27.24	0.57	23.32	75.6	1.70	–
**IL-1β**	0.17	7.12	5.40	0.56	23.92	5.41	6.53
**IL-1α**	<0.16*	0.94	2.58	0.72	3.54	2.29	3.36
**IL-1RA (*IL-1RN*)**	50	268.80	2.43	77.6	456	2.55	2.99
**TNFα**	<0.06*	101.20	≥10.70	0.29	145.2	8.97	4.78
**IL-10**	0.27	57.60	7.71	0.66	44.4	6.08	4.70
**GM-CSF (*CSF2*)**	<0.065*	7.32	≥6.81	<0.065*	29.32	≥8.81	7.81
**TSLP**	<0.056*	0.97	≥4.10	0.056*	14.48	≥8.00	–
**IL-6**	9.28	116	3.64	11.76	367.2	4.96	3.47
**IL-12/23p40 (*IL-12B*)**	<0.06*	0.57	≥3.15	<0.06*	5.92	≥6.53	–
**IL-13**	<0.36*	7.00	≥4.92	0.36*	36.56	≥6.68	–
**IL-27**	1604	1648	0.039	2800	2180	-0.36	0.14
**bFGF (*FGF2*)**	11.52	10.08	-0.19	9.40	7.96	-0.24	–
**Flt-1 (*VEGFR1*)**	1464	1472	0.0079	3012	2752	-0.13	–
**IL-28B (*IFNλ3*)**	149.37	185.44	0.31	174.37	181.93	0.06	5.60

Cells were infected (+Lm), or not (NI), for a total of 5 and 10 h as in RNAseq experiments. The cell culture supernatants were collected, centrifuged and immediately and frozen. Cytokine production was measured using the V-PLEX Human Biomarker 54-Plex Kit (Meso Scale Discovery). Samples from 3 independent experiments were pooled and analyzed in duplicates, results are presented as the average cytokine concentration and fold change (FC) between infected and non-infected samples. IFNλ3 was measured by ELISA. (*): (Value < detection limit). (-): transcript below threshold of having an expression level of 2 counts per million in at least half of the samples in RNAseq.

### *L. monocytogenes* Induces Cytokine and Chemokine Production in Both Syncytia and Mononuclear Trophoblasts, but With Distinct Prevalence

**Figure 6:**
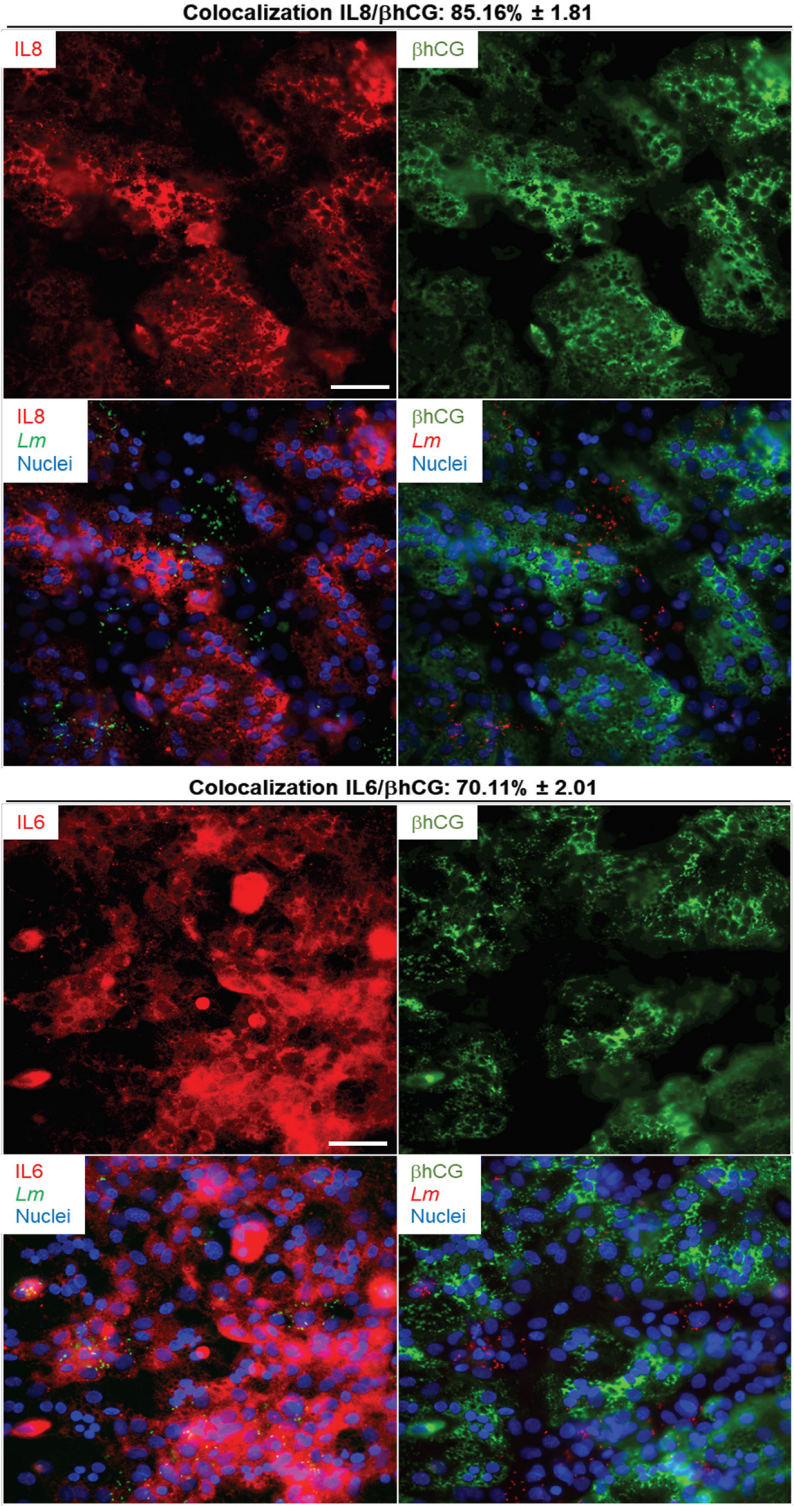
IL-6 and IL-8 colabeling with βhCG in infected primary trophoblasts. PHT were infected with p*actA*-RFP *L. monocytogenes* (MOI 1) for 5 h using the same experimental procedure as the RNAseq infection experiments. Cells were fixed, permeabilized and labeled for IL-6 (mouse primary Ab and secondary Ab-Alexa Fluor 647), IL-8 (mouse primary Ab and secondary Ab-Alexa Fluor 647), βhCG (rabbit primary Ab and secondary Ab-Alexa Fluor 488), and nuclei were labeled with DAPI. At least thirty fluorescent images were randomly acquired with the 40X objective. Representative images are from one experiment. Scale bar = 50 µm. The Metamorph software was used to measure the percent colocalization between each cytokine and, βhCG the average colocalization ± SEM is indicated at the top of the images.

**Figure 7:**
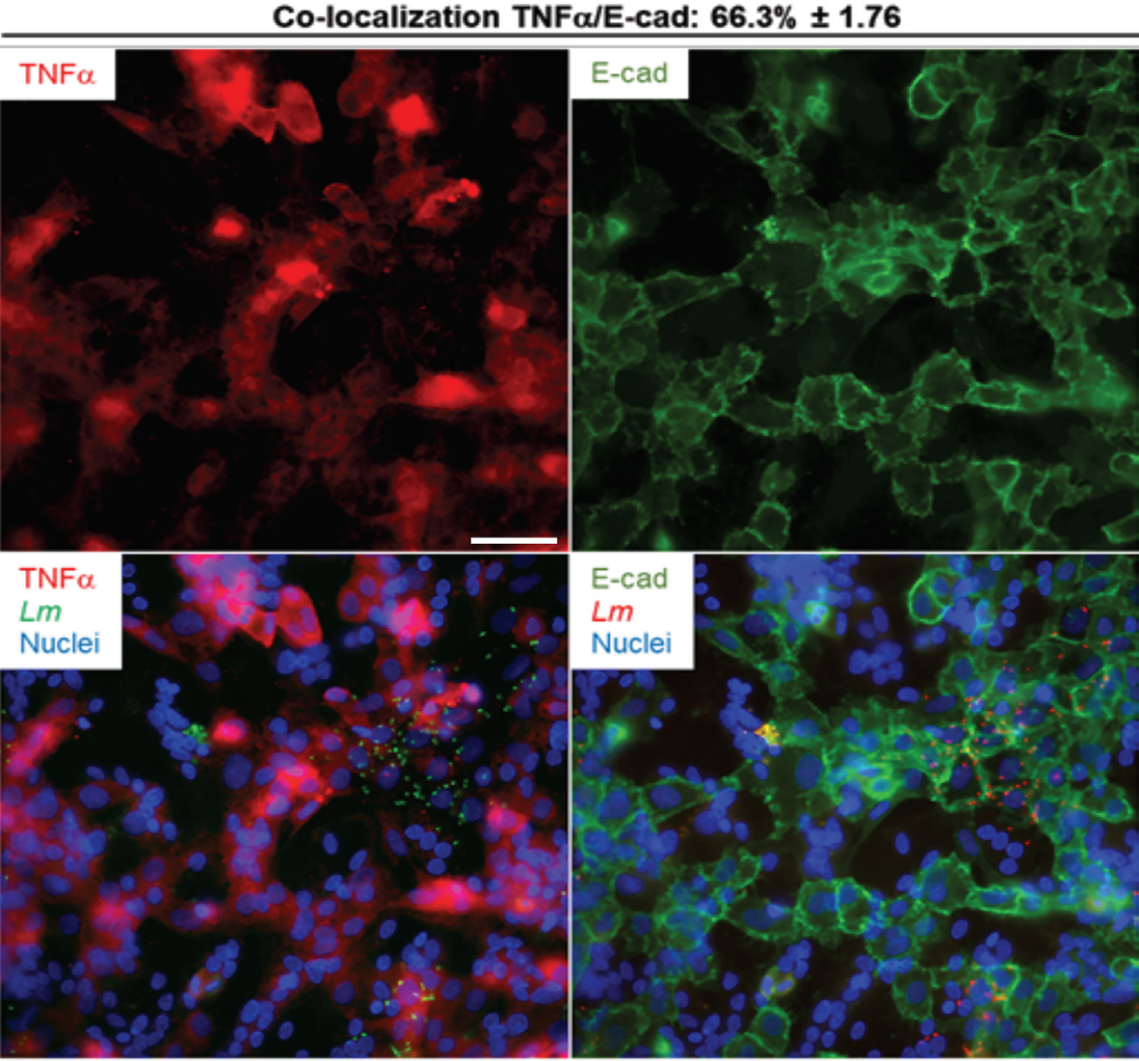
TNFα colabeling with E-cadherin in infected primary trophoblasts. PHT were infected with p*actA*-RFP *L. monocytogenes* (MOI 1) for 5 h using the same experimental procedure as the RNAseq infection experiments. Cells were fixed, permeabilized and labeled for TNF-α (rabbit primary Ab and secondary Ab-Alexa Fluor 647) and for E-cadherin (mouse primary Ab and secondary Ab-Alexa Fluor 488). At least thirty fluorescent images were randomly acquired with the 40X objective. Representative images are from one experiment. Scale bar = 50 µm. The MetaMorph software was used to measure the percent colocalization between TNFα and E-cadherin, the average colocalization ± SEM is indicated at the top of the images.

We next established if the inflammatory mediators detected at the transcriptional and translational levels in infected PHT were expressed by both syncytia and non-fused trophoblasts, and if infected cells were exclusively responsible for expression of these mediators. Cells were infected with RFP-expressing *L. monocytogenes* for 5 h (same experimental conditions as in RNAseq). Cells were fixed, permeabilized, and fluorescently labeled for the cytokines (IL-6, IL-8, or TNFα), and βhCG or E-cadherin (**Figures 6**, **7** and **S7**). E-cadherin and βhCG were selected as prototypical cell fusion markers since cell fusion is accompanied by loss of E-cadherin expression and gain of βhCG expression (15, 82). As a control, we performed E-cadherin and βhCG colabeling in PHT, confirming the segregation of these two fusion markers (**Figure S8**). We selected IL-6, IL-8, and TNFα as their production was markedly enhanced post-infection (**Table 4**). IL-8 was produced at basal level and its expression was enhanced upon infection, while the two others (IL-6, and TNFα) were mostly induced by *L. monocytogenes*. As expected, we found that *L. monocytogenes* predominantly infected non-fused PHT (E-cadherin-positive and βhCG-negative PHT) (**Figures 6**, **7**). There was no correlation between cell infection and their cytokine expression level, as cytokines were detected in similar amount in both infected and non-infected cells (data not shown). We found that syncytia and non-fused trophoblasts both expressed the three cytokines. However, there was a correlation between the cell fusion status and the pattern of cytokine expression. Indeed, IL-8 strongly colocalized with βhCG-producing cells (∼85%), which means that 85% of the total IL-8 fluorescence intensity was associated with fused PHT. IL-6 also colocalized with fused cells, but to a lesser extent than IL-8 (∼70%). On the contrary, TNFα was expressed more evenly between fused and non-fused cells, with some enrichment (∼66%) in non-fused PHT (E-cadherin expressing PHT). In conclusion, our data support that the transcriptional pathways analyzed by RNAseq are representative of both syncytia and non-fused trophoblasts and that PHT expressed pro-inflammatory cytokines independently of their fusion and infection status (at least for IL-6, IL-8, and TNFα). Interestingly, we show for the first time that syncytia and non-fused trophoblasts appear to be more portent at producing some cytokines than others in response to exposure to *L. monocytogenes*.

## Discussion

Trophoblasts are epithelial cells located at the maternal/fetal interface that play key immune roles by protecting the fetus from rejection by the maternal immune system and by forming an anti-infective barrier (12, 19, 83). We still have limited knowledge about the trophoblast antimicrobial and inflammatory roles during infection by *L. monocytogenes*. Our studies are in agreement with the notion that trophoblasts act as placental gatekeepers that limit *L. monocytogenes* infection, and act as immune sensors of infection. In response to infection by *L. monocytogenes*, trophoblasts produce pro-inflammatory cytokines (predominantly IL-6 and TNFα) as well as neutrophil, natural killer, and monocyte chemokines (predominantly IL8 and MIP-1α/β). In parallel, trophoblasts maintain or increase the production of cytokines such as IL-27, IL1-RA and IL-10, which are expected to balance the activation of the pro-inflammatory innate immune response with the suppression of the maternal anti-fetal adaptive immunity (79–81). However, a collection of upregulated cytokines and other inflammatory genes observed in our studies are associated with infection of the placental-fetal unit and pregnancy complications such as chorioamnionitis and preterm birth. These observations suggest that if *L. monocytogenes* is not cleared from the placenta, trophoblast responses are likely to become deleterious to pregnancy.

We found that syncytia of primary human trophoblasts and BeWo cells are about 20-fold more resistant to infection than non-fused trophoblasts. This is in accordance with previous reports showing that fusion of murine trophoblasts increased their resistance to *L. monocytogenes* by 25-fold and the SYN of human placental explants displayed low susceptibility to this pathogen (10, 70, 84). We propose for the first time that the SYN barrier function operates *via* blocking initial bacterial entry rather than limiting bacterial phagosomal escape or intracellular proliferation (**Figures 1**–**4** and **S2**). The loss of *L. monocytogenes* association to the SYN is explained by its faint level of E-cadherin expression, the receptor for the bacterial surface invasin InlA (**Figures 1A, B**, **2** and **S2**) (8, 38).

Elucidating the roles of InlA and InlB in infection of the placental/fetal unit has been challenging due to the species-specificity of InlA and InlB. *In vivo* animal studies using E-cadherin-humanized mice and gerbils (permissive to InlA- and InlB-mediated invasion), *ex vivo* analysis of human placental explants, and epidemiological studies, support that InlA is important for placental infection (10, 39, 40, 85). Other studies using the guinea pig model (permissive to InlA, but not InlB), or an InlA-murinized *L. monocytogenes* strain in an oral murine infection model (permissive to InlB, but not InlA), concluded that InlA is dispensable for *L. monocytogenes* transmission to the placental/fetal unit (36, 38, 86). The discrepancies between these studies might be explained by differences in the species specificity of InlA and InlB, the infectious route, and by the bacterial strains used in these studies. Indeed, it was proposed that the role of InlA in crossing the placental barrier *in vivo* requires the conjugated action of InlA and InlB, which entry pathways are simultaneously functional in the murine humanized and gerbil models, but not in guinea pigs (87). However, the bacterial strain mostly used in these studies carries a mutation in the transcriptional regulator of virulence genes *prfA* leading to elevated expression of InlA/InlB (57). Also, mInlA, expressed by the InlA-murinized *L. monocytogenes* strain, was shown to bind to other receptors in addition to E-cadherin, which questions the relevance of this strain (37). It was proposed that InlA may contribute to low level of infection of the SYN (8) and/or to infection of other cell types that express higher levels of E-cadherin. These could be maternal cells that are infected before the bacterium reaches the placental/fetal unit, or placental endovascular trophoblasts and extravillous trophoblasts, which are present at the maternal/fetal interface (10). Also, it was proposed that *L. monocytogenes* could invade subsyncytial trophoblasts, expressing high E-cadherin levels, at locations where the SYN is damaged (10).

We did not identify a role for InlB in *L. monocytogenes* uptake by human trophoblasts using the *L. monocytogenes* strain 10403S [(71) and this work]. Similarly, other studies using the strain 10403S, made the same conclusion (10, 36). This is most likely due to the low level of InlB expressed by 10403S, as we and others have previously shown (57, 71, 88). Indeed, studies that used *L. monocytogenes* strains expressing higher InlB levels identified a role for InlB in invasion of cultured trophoblasts and in placental infection *in vivo* (39, 71, 89, 90).

The *L. monocytogenes* virulence factor ActA was previously shown to facilitate bacterial uptake by Caco-2, HeLa, MDCK, and Vero epithelial cells (44). Our study identified a previously unrecognized role for ActA in facilitating *L. monocytogenes* internalization into primary mononuclear trophoblasts, but not in syncytia (**Figure 2E**). The nature of the molecules involved in ActA-mediated *L. monocytogenes* internalization and how these molecules are altered in syncytia are unknown.

ActA most importantly promotes *L. monocytogenes* cytosolic motility for cell-to-cell spread and is required for placental-fetal infection of various small animals (91). Remodeling of the F-actin cytoskeleton in the SYN suggested that the *L. monocytogenes* intracellular motility could be altered (15). However, we found that the average percentage of motile *L. monocytogenes* and their average speed were similar in the SYN and non-fused trophoblasts (**Figure 4C**). A study using a murine SYN model, proposed that the high rigidity of the subcortical actin cytoskeleton in the SYN decreased *L. monocytogenes* cell-to-cell spreading to these cells (70). In accordance with these latter finding, we observed that fused cells in proximity to infectious foci of non-fused cells most often remained non-infected.

The bacterial cytosolic doubling time, measured between 1 and 5 h and between 5 and 7 h of infection, were similar in fused and non-fused trophoblasts (**Table 1** and **Figure 3**). Together with cell invasion experiments (**Figures 2** and **S2**), these data support that the anti-*L. monocytogenes* barrier function of the SYN primarily consists in preventing bacterial entry and not the subsequent intracellular proliferation, at least during the first 7 h of infection.

Interestingly, we found that intracellular doubling time of *L. monocytogenes* increased in trophoblasts from ~ 70 min (1-5 h post-infection) to 200 min (5-7 h post-infection). The underlying mechanisms are currently unknown, do not involve the antibiotic gentamicin, and are not specific to trophoblasts. This appears to be a general feature, as *L. monocytogenes* doubling time was 180 min in MDCK infected for 6 to 22 h (92). We observed a population of *L. monocytogenes* in PHT (fused and non-fused) that were unable to nucleate the host cell actin, and the elongation of *L. monocytogenes* in PHT at late infection times (≥ 10 hours) (**Figure S4**). However, we found that bacterial elongation was influenced by the presence of gentamicin, which does not preclude that PHT may also limit bacterial growth at late infection times.

Previous studies analyzing primary human extravillous trophoblasts (first trimester pregnancy) showed that *L. monocytogenes* failed to escape the phagosome and to replicate in these cells (11). Therefore, extravillous trophoblasts, which express high E-cadherin levels, are more permissive to initial *L. monocytogenes* uptake, but then restrict intracellular bacterial proliferation, a property not shared with third trimester SYN and sub-syncytial trophoblasts (our study). In addition, decidual natural killer cells, which are in close proximity to extravillous trophoblasts at the chorionic villi anchoring sites, were recently shown to transfer granulysin into infected extravillous trophoblasts leading to the restriction of *L. monocytogenes* cytosolic growth (93).

We report the first RNAseq analysis of *L. monocytogenes*-infected trophoblasts. Trophoblast exposure to *L. monocytogenes* mainly resulted in the upregulation of 330 protein-coding genes. Gene enrichment analysis revealed that gene upregulation likely involves transcriptional activation *via* RNA polymerase II (**Figures 5C, D** and **Table 3**). The upregulated genes were predominantly related to immunity, inflammation, signaling, and transcription. Genes were enriched in pro-inflammatory pathways activated by pattern recognition receptors (PRRs) such as TLR2 and cytosolic receptors, and *via* autocrine stimulation by cytokines such as TNFα (**Table 3** and **Figures S5**, **S6**). Several studies showed that trophoblasts express various pattern recognition receptors to detect and respond to pathogens; thereby playing a role in placental immunity (19, 94). In particular, we found that the transcripts encoding TLR2, which recognizes bacterial lipoproteins, peptidoglycan, and lipoteichoic acid (95–98), RIG-I (DDX58) and MAD-5 (IFIH1), which are involved in the cytosolic detection of *L. monocytogenes* nucleotides (99, 100), were upregulated upon *L. monocytogenes* infection. Also, trophoblasts including the SYN are known to express TLR2 (**Figure S5**) (101). The immune signaling pathways involve the MAP kinases and JAK-STAT, among others, and converge toward NF-κB activation and other transcription factors (**Table S3**) leading to transcriptional activation of a collection of cytokines and chemokine genes (**Figures S5**, **S6**). Gene-disease mappings using the “Genetic Association Database” highlighted common genes enriched in viral infections. At 5 h post-infection, infected or non-infected PHT, produced TNFα, IL-8 and IL-6 (**Figures 6**, **7**). Together, these observations support that trophoblasts detected both extracellular and intracellular bacteria and mounted a pro-inflammatory response resulting in the release of inflammatory mediators (**Table 4**), which could stimulate neighboring cells. Importantly, there was congruence between the fold changes of transcripts and corresponding cytokines and chemokines detected in the trophoblast culture supernatants. For example, *L. monocytogenes* increased the production of MIP-1α/β (100- to 1000-fold), IL-8 (70-fold), TNFα (~1000-fold), IL-6 (30-fold), IL1-RA (6-fold), IL-1α/β (6- to 30-fold), GM-CSF (250-fold), IL-13 (100-fold), and IL-10 (200-fold). Trophoblasts isolated from term placentas have been previously shown to produce MIP-1α and MIP-1β along with IL-6, IL-8, IL-1β, TNFα, GM-CSF (*csf2*) (29, 102). Unlike primary cells, neither the BeWo transcriptome nor release of mediators were affected in a significant fashion upon *L. monocytogenes* infection, and therefore cannot be used as a trophoblast surrogate to study immune gene regulation downstream to *L. monocytogenes* infection.

Infection of small animal models by *L. monocytogenes* showed that neutrophils and other maternal leukocytes were massively recruited in the placenta (103). Our transcriptomic and cytokine data support that trophoblasts participate in the recruitment and activation of maternal leukocytes, neutrophils primarily, but also monocytes and T lymphocytes *via* the production of chemokines such as IL-8, MIP-1α and MIP-1β. The basal secretion of IL-8 (**Table 4**) and the predilection of the SYN for the production of this cytokine (**Figure 6**) are in accordance with elevated IL-8 levels in the SYN of healthy placentas (104, 105). TNFα plays an important role in *L. monocytogenes* immunity (106, 107). In mice infected with *L. monocytogenes*, TNFα was produced early in infection (108) and neutralization of TNFα exacerbated listeriosis (108). However, TNFα can induce trophoblast apoptosis (109). We did not observe any apoptotic cells based on nuclear structures observed by DAPI fluorescence for up to 24 h infection. RNAseq analysis showed the upregulation of 25 transcripts of anti-(BIRC2, BIRC3, FLIP, XIAP, SOCS3, PLAUR, etc.) and pro-(TRAILR2, BCL2, TNFRSF10A) apoptotic genes. However, pathway analysis revealed a negative regulation of apoptosis indicating that, together with the absence of apoptotic trophoblasts, anti-apoptotic signals prevailed. IL-6 is required for the control of *L. monocytogenes* in a non-pregnant murine model (110), therefore IL-6 production by trophoblasts may also have beneficial protective effects in placental immunity. As an important first line of defense, we found the transcripts for IL-1β, IL-18, GBP5, and caspase-1 to be significantly upregulated, supporting recent work showing that *L. monocytogenes* infection of placental villi activates the NLRP3 inflammasome, which provides placental anti-microbial protection (84). Accordingly, several studies initially reported NLRP3 stimulation by *L. monocytogenes* (111, 112) and barrier cells such as the intestinal epithelium and trophoblast cells produce the NLRP3 inflammasome (113, 114). However, activation of the NLRP3 inflammasome and IL-1 production can lead to embryo loss in *L. monocytogenes* infected mice (111). We found that trophoblasts produce IFNλ at basal level, as reported by others (78). Although the genes encoding IFNλs were all upregulated upon *L. monocytogenes* infection, we did not observe an increase in proteins for up to 24 h post-infection (**Table S5**). Of note, a study reported IFNλ production by epithelial cell lines infected by *L. monocytogenes*, but used a very high MOI of 50 to 100 for over 24 h (77). Therefore, increased IFNλ production may require high *L. monocytogenes* infection levels and prolonged infection times.

The placenta must tightly control inflammation to tolerate the semi-allogeneic fetus while maintaining host defense. Modulation of the immune response based on the microorganism is necessary to maintain growth of the fetus and limit pathogen invasion (115). In that respect, trophoblasts are unlike any other epithelial cells due to their tolerogenic functions (19). Interestingly, IL-27 is constitutively produced by trophoblasts and unaffected by *L. monocytogenes* infection (**Table 4**). IL-27 is known to promote T cell tolerance and modulate TLR expression (79, 116, 117). IL-10 is also an important anti-inflammatory cytokine that contributes to T cell tolerance, and its production was increased 200-fold 5 h post-infection upon *L. monocytogenes* infection (**Table 4**). Similarly, the IL-1 antagonist IL1-RA and the trophoblast-derived thymic stromal lymphopoietin (TSLP) production were increased upon *L. monocytogenes* infection (**Table 4**) (84). For infections such as listeriosis, for which sterilizing immunity requires T cell immunity, it is thought that the suppression of placental T cell responses for fetal tolerance is responsible for sustained infection of the placental-fetal unit despite the initial innate immune response. However, as the pathogen persists, placental inflammation may override suppression of maternal allo-reactive T cell responses. Indeed, bacterial and viral infections have been strongly associated with pregnancy disorders such as pre-eclampsia, intrauterine growth retardation, and spontaneous abortion (118). The fact that transcriptome of infected trophoblasts includes upregulation of signature genes involved in placental inflammation and preterm birth supports that the trophoblast pro-inflammatory responses may become deleterious. In conclusion, infected trophoblasts are balancing their pro-inflammatory activity by releasing tolerogenic factors in accordance with their known function of immune suppressor or of T cell activation. It is likely, that the initial inflammatory response of trophoblasts may have beneficial effects by recruiting and activating maternal neutrophils and monocytes, and by activation of NLRP3, but if infection is not cleared, prolonged and exacerbated production of pro-inflammatory mediators by trophoblasts may favor the known poor pregnancy outcomes of listeriosis.

## Data Availability Statement

The original contributions presented in this study are publicly available. The data have been deposited in NCBI's Gene Expression Omnibus (119) and are accessible through GEO Series accession number GSE175815 (https://www.ncbi.nlm.nih.gov/geo/query/acc.cgi?acc=GSE175815).

## Ethics Statement

The studies involving human participants were reviewed and approved by The Ohio State Biomedical IRB (protocol #2017H0478) and the Office of Responsible Research Practices. The patients/participants provided their written informed consent to participate in this study.

## Author Contributions

LJ, SA, and JM carried out experiments. LJ performed numerical calculations and analyzed the data. AW processed RNAseq data. XZ consulted on statistical analysis. MG supervised RT-qPCR experiments. KR obtained placentae. LJ and SS wrote the manuscript with input from all other authors. SS supervised the project. All authors contributed to the article and approved the submitted version.

## Funding

Research reported in this publication was supported by the Institute of Allergy and Infectious Diseases of the National Institutes of Health under award numbers R21AI105588, R03AI149371, and R01AI157205 and by funds from the Center for Clinical and Translational Science at The Ohio State University (CTSA grant number UL1TR001070). This work was sponsored by NIH/NIAID award #1-T32-AI-112542, a NRSA training grant administered by the Center for Microbial Interface Biology at The Ohio State University (postdoctoral fellowship).

## Author Disclaimer

The content is solely the responsibility of the authors and does not necessarily represent the official views of the National Institutes of Health.

## Conflict of Interest

The authors declare that the research was conducted in the absence of any commercial or financial relationships that could be construed as a potential conflict of interest.

## Publisher’s Note

All claims expressed in this article are solely those of the authors and do not necessarily represent those of their affiliated organizations, or those of the publisher, the editors and the reviewers. Any product that may be evaluated in this article, or claim that may be made by its manufacturer, is not guaranteed or endorsed by the publisher.
