# Supplementary figures and images for: Human Placental Trophoblasts Infected by Listeria monocytogenes Undergo a Pro-Inflammatory Switch Associated With Poor Pregnancy Outcomes
Source: Front Immunol. 2021 Jul 23;12:709466. doi: 10.3389/fimmu.2021.709466 (PMC8346206; doi:10.3389/fimmu.2021.709466)

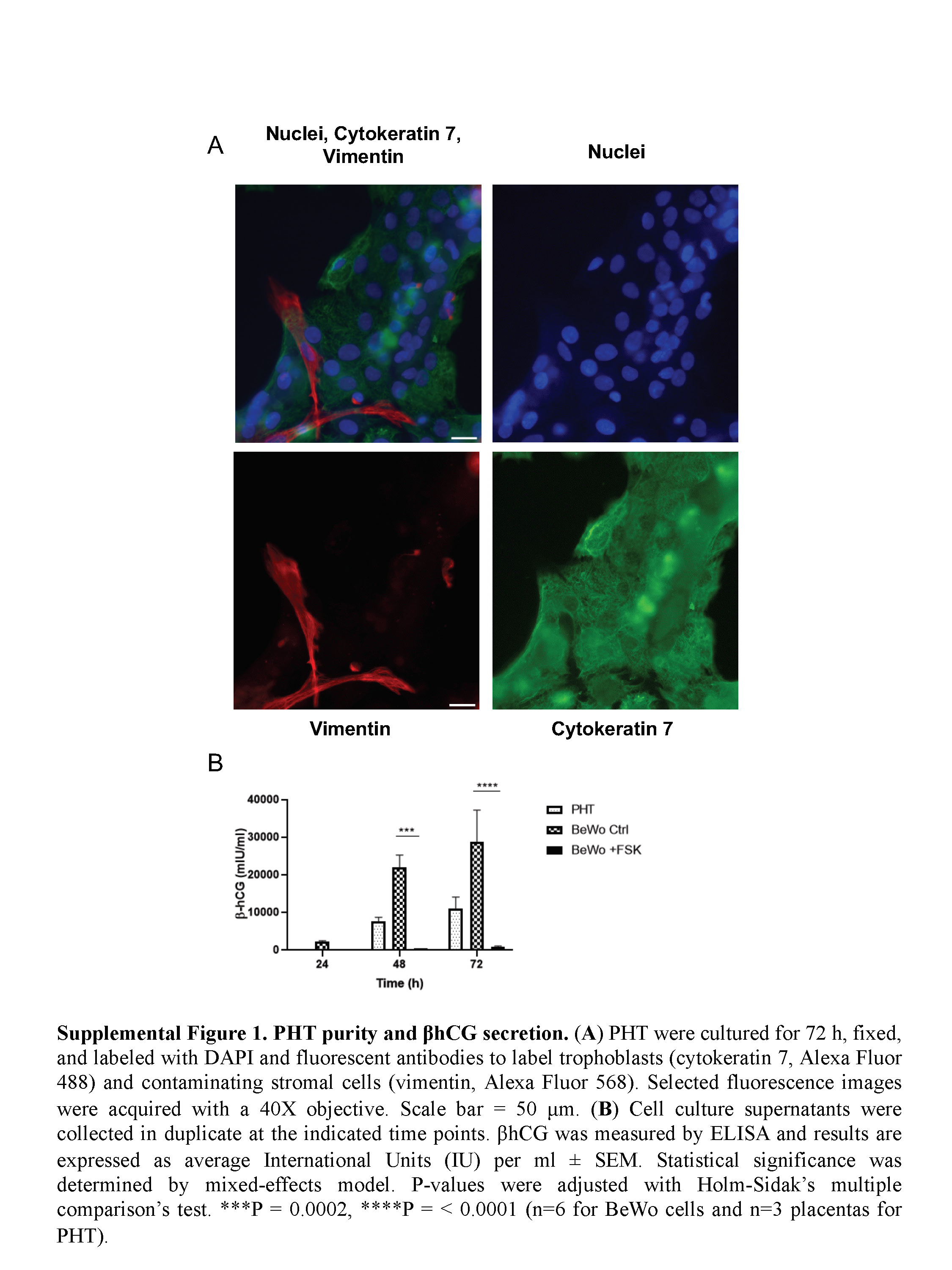

Supplement: Supplementary file 1 [file Image_1.jpeg]

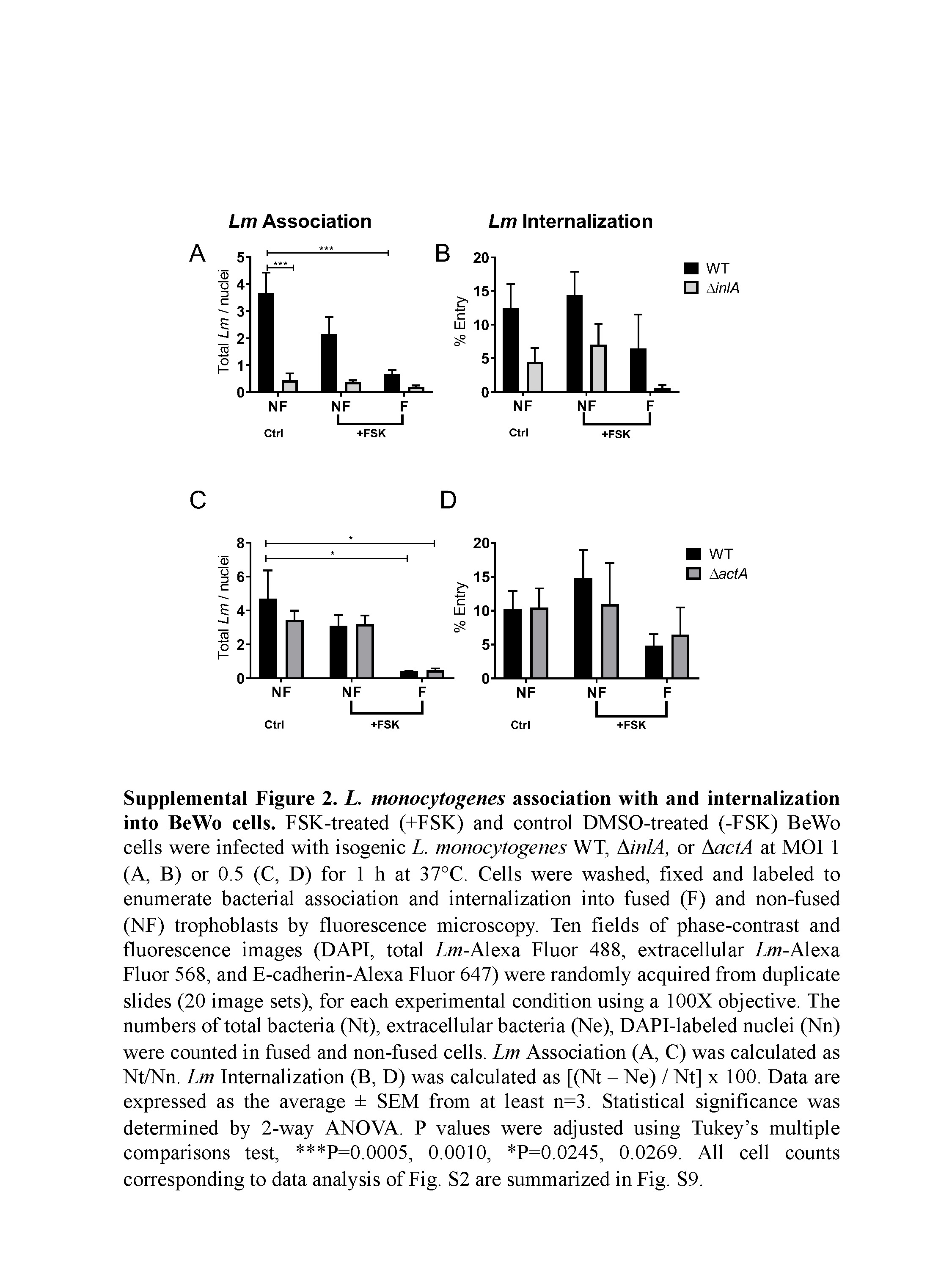

Supplement: Supplementary file 2 [file Image_2.jpeg]

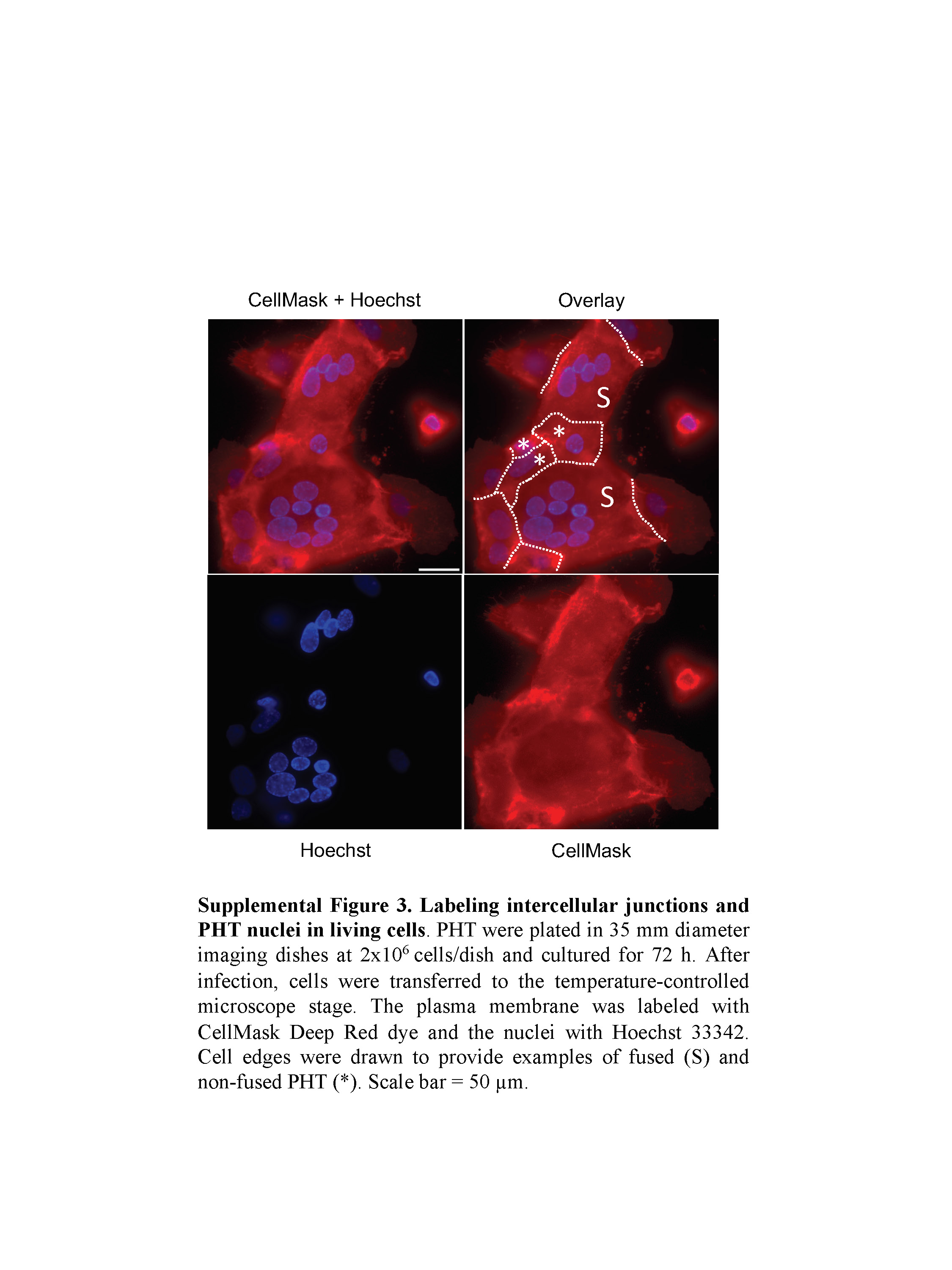

Supplement: Supplementary file 3 [file Image_3.jpeg]

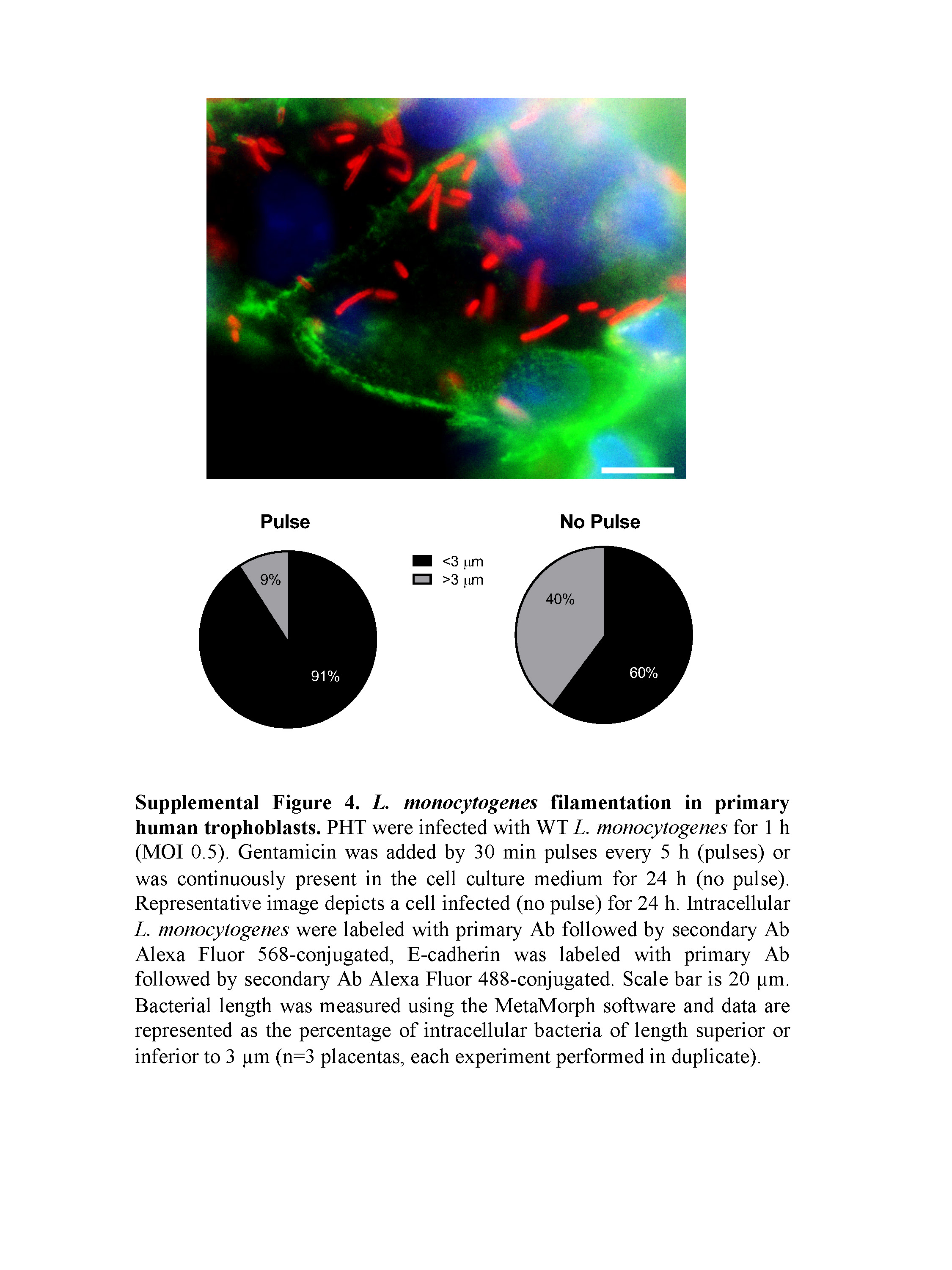

Supplement: Supplementary file 4 [file Image_4.jpeg]

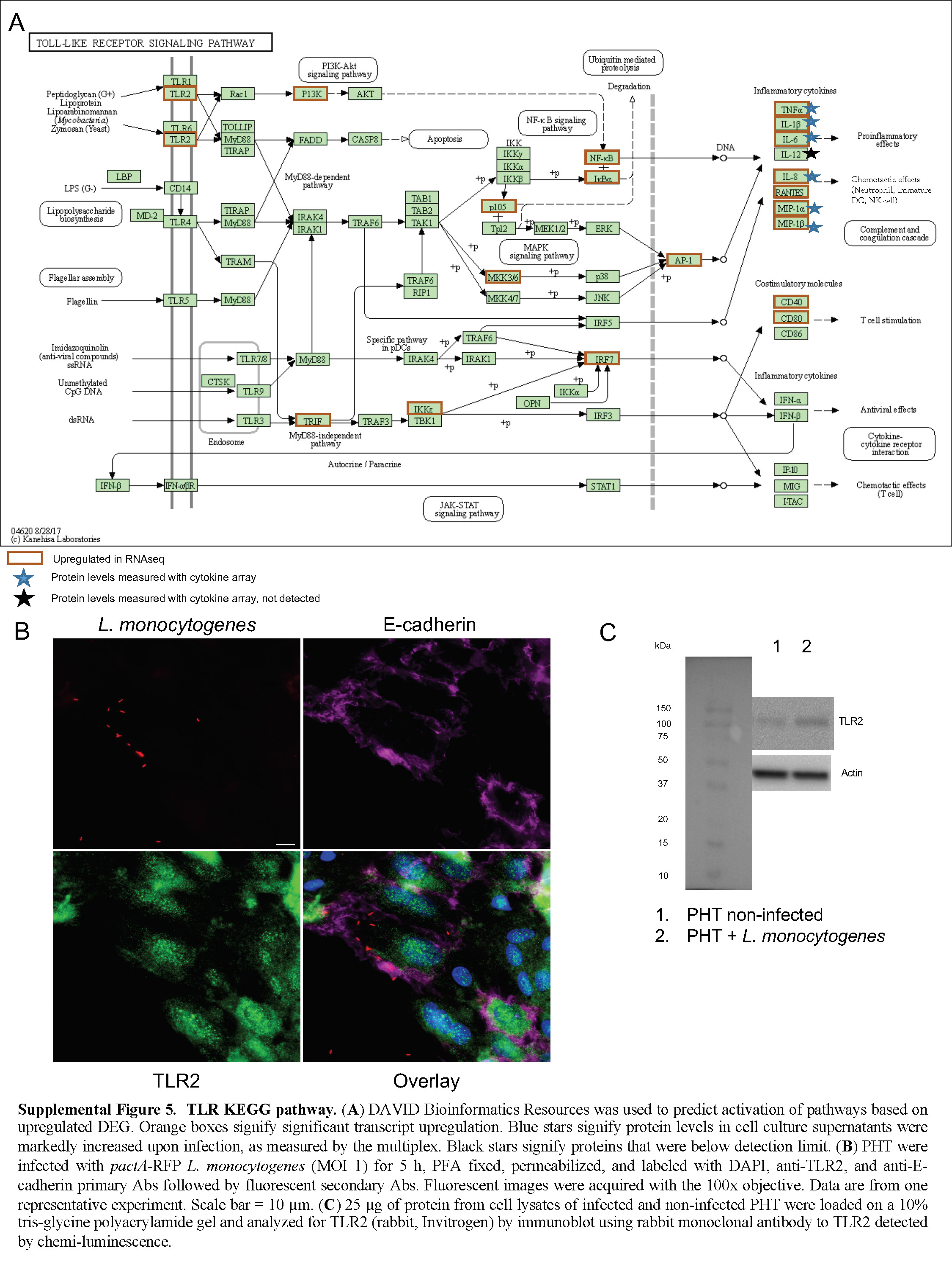

Supplement: Supplementary file 5 [file Image_5.jpeg]

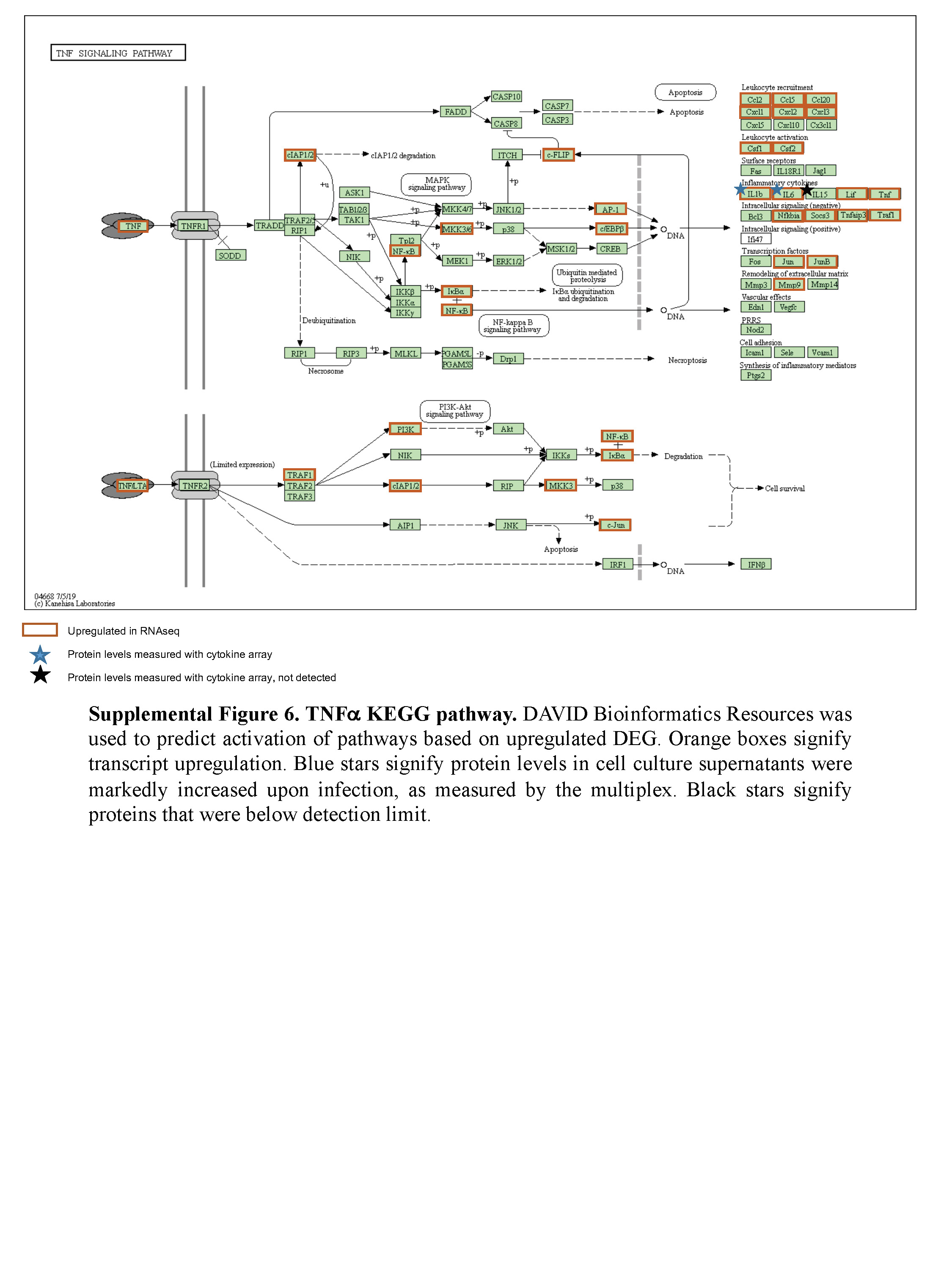

Supplement: Supplementary file 6 [file Image_6.jpeg]

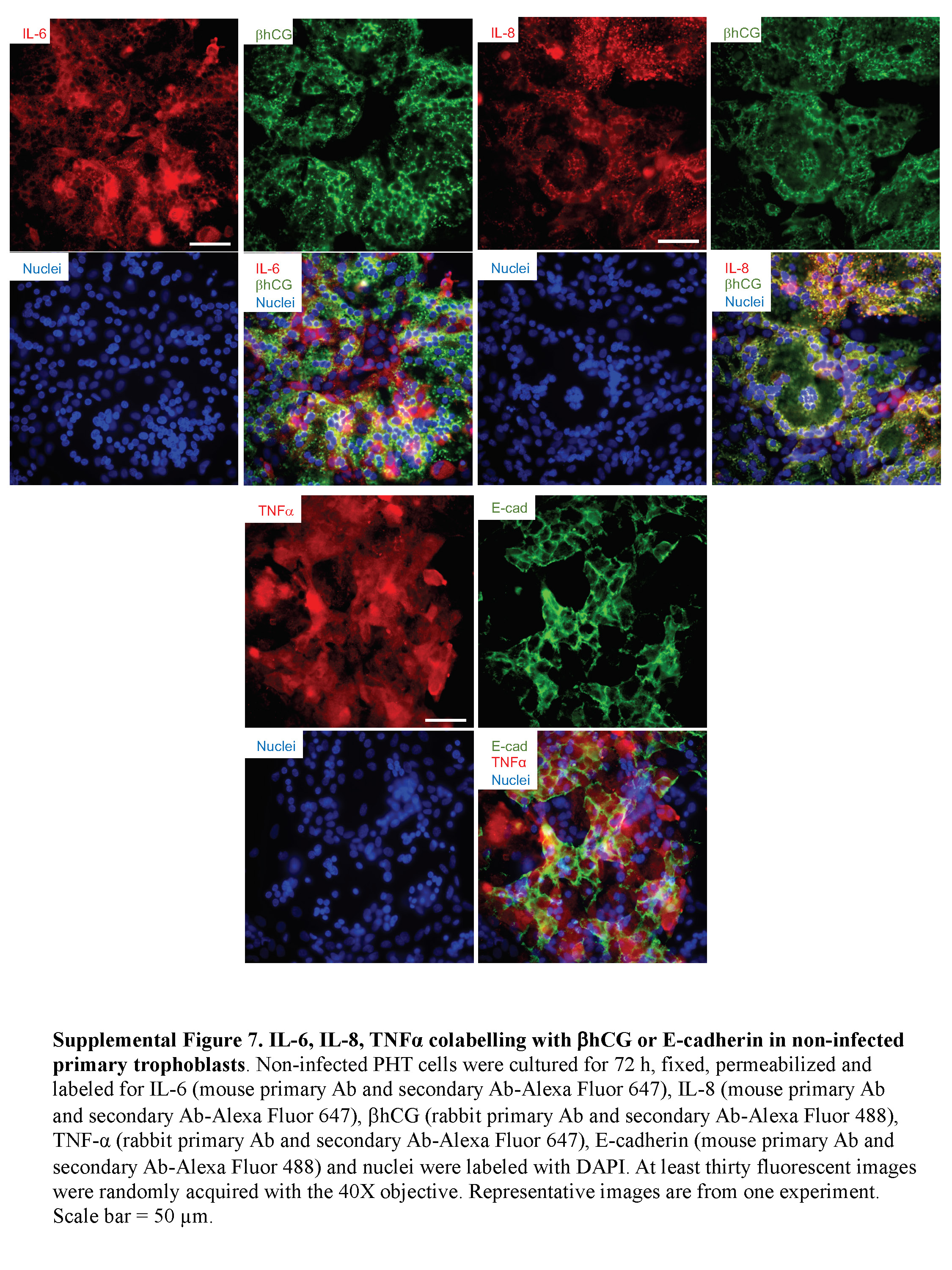

Supplement: Supplementary file 7 [file Image_7.jpeg]

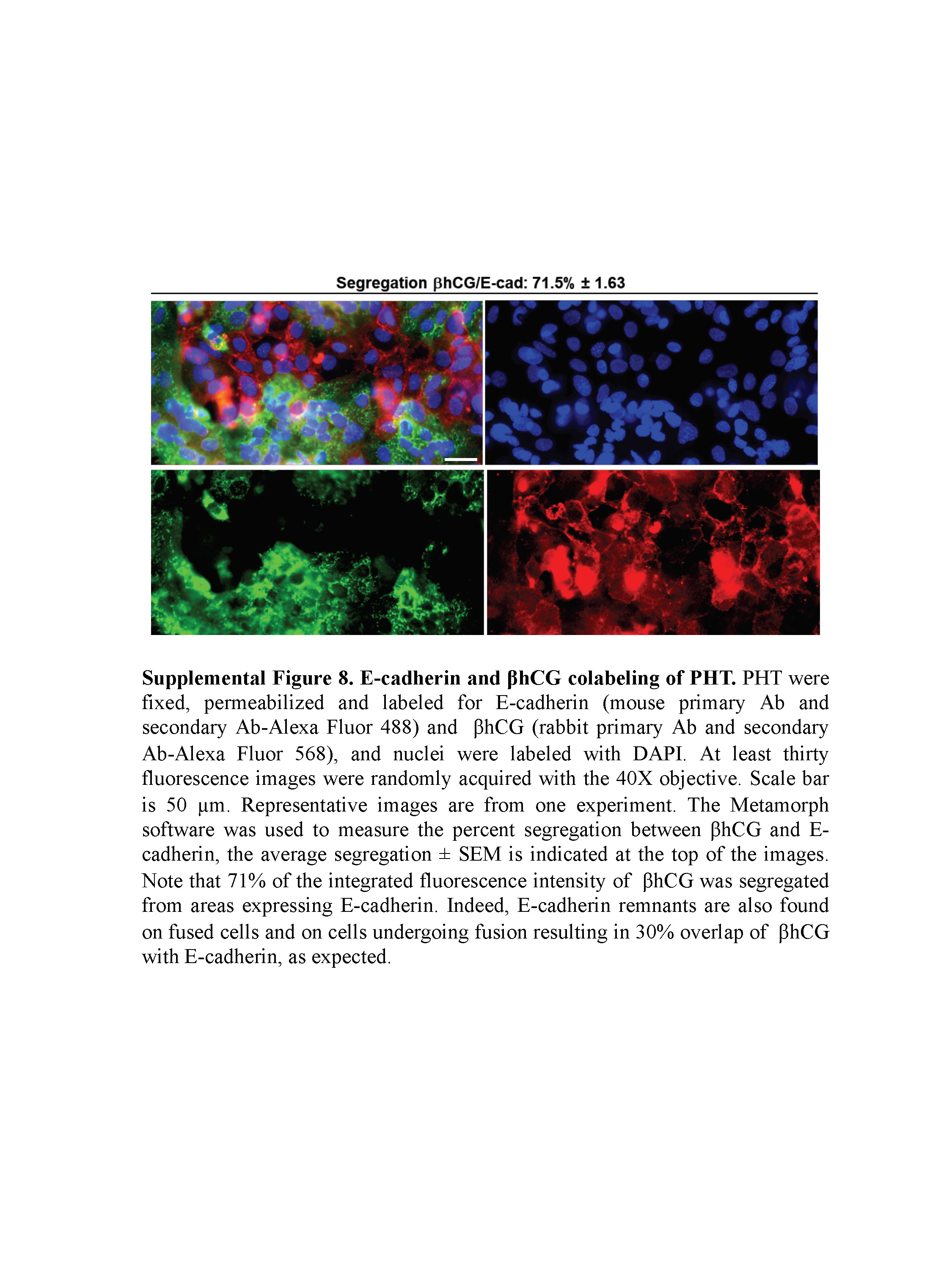

Supplement: Supplementary file 8 [file Image_8.jpeg]

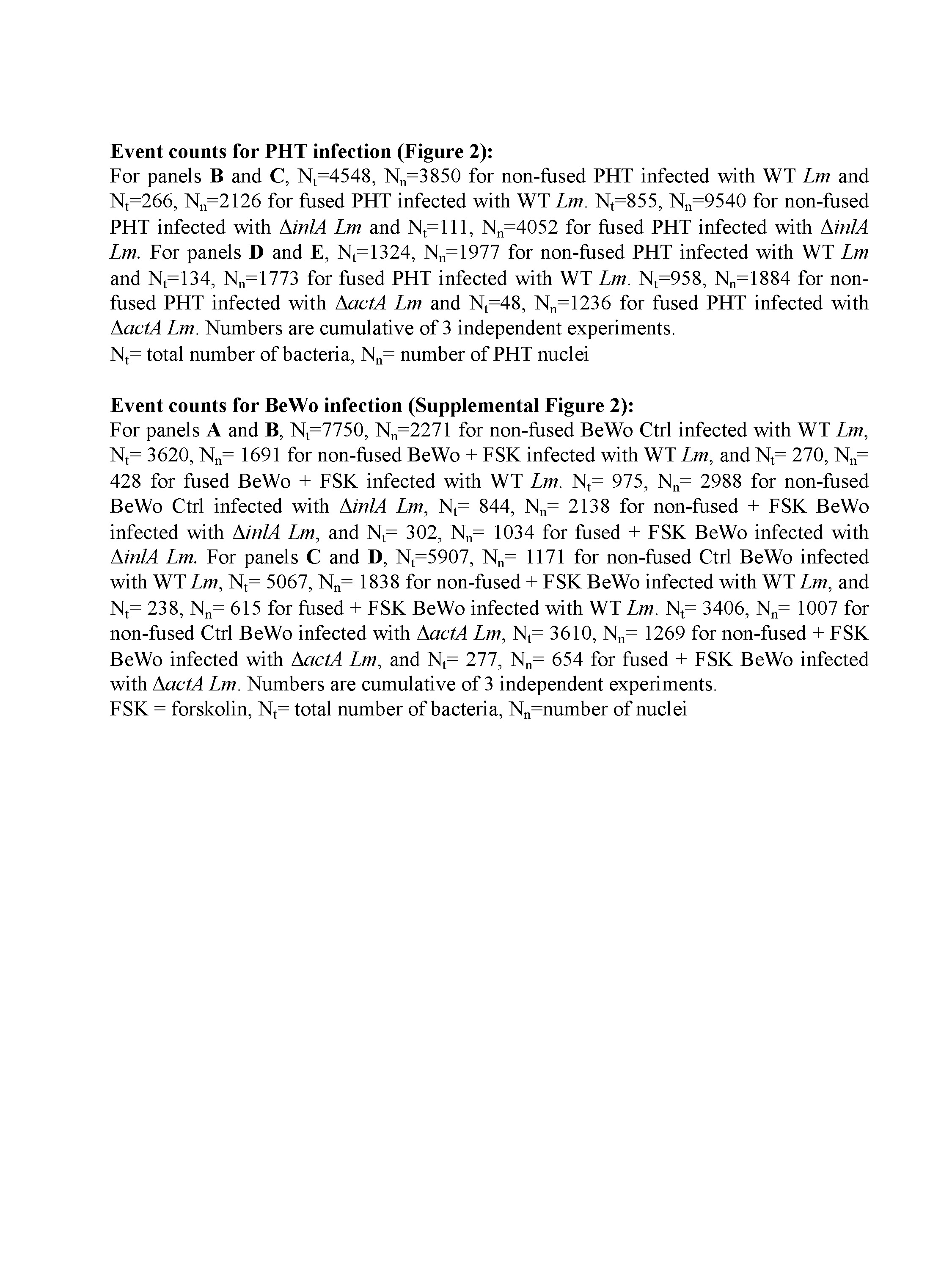

Supplement: Supplementary file 9 [file Image_9.jpeg]
