# Supplementary material for: Human Placental Trophoblasts Infected by Listeria monocytogenes Undergo a Pro-Inflammatory Switch Associated With Poor Pregnancy Outcomes
Source: Front Immunol. 2021 Jul 23;12:709466. doi: 10.3389/fimmu.2021.709466 (PMC8346206; doi:10.3389/fimmu.2021.709466)
Supplement: Supplementary file 10 [file Table_1.docx]

|  | **RNAseq** | | | **qPCR** | | |
| --- | --- | --- | --- | --- | --- | --- |
| **Gene** | **Log_2_(CPM) *Lm*** | **Log_2_(FC)** | **FDR** | **RCN**  ***Lm*** | **Log_2_ (FC)** | **P-value** |
|  |  |  |  |  |  |  |
| ***IFNλ1*** | 1.25 | 5.54 | 2.30E-22 | 1.25 | 3.70 | 0.03 |
| ***IFNλ2*** | 1.84 | 5.51 | 5.22E-15 | 1.02 | 6.30 | 0.01 |
| ***TNF*** | 5.45 | 4.78 | 1.69E-45 | 16.39 | 6.63 | 0.01 |
| ***IL-8*** | 9.06 | 4.17 | 3.81E-19 | 603.07 | 5.25 | 0.00001 |
| ***IL-1β*** | 7.62 | 6.53 | 3.03E-64 | 106.37 | 8.83 | 0.001 |
| ***IL-6*** | 4.98 | 3.47 | 5.79E-28 | 32.80 | 4.45 | 0.001 |
| ***IL-10*** | 1.84 | 4.70 | 3.80E-11 | 2.03 | 5.67 | 0.114* |

**Supplemental Table 1: RT-qPCR validation of RNAseq (PHT).** A collection of cytokine-coding genes differentially expressed upon infection were selected to confirm RNA-seq data by RT-qPCR. RT-qPCR data are the average of 3 independent experiments performed with 3 different placentas (different from placentas used for RNAseq). FDR is represented as value ± standard error of the mean. CPM (counts per million) value for *Lm*-infected samples; FC (Fold change) compares non-infected to infected samples; FDR (false discovery rate); RCN (relative copy number) value of the *Lm* infected samples. * = not significant
