# Supplementary material for: Human Placental Trophoblasts Infected by Listeria monocytogenes Undergo a Pro-Inflammatory Switch Associated With Poor Pregnancy Outcomes
Source: Front Immunol. 2021 Jul 23;12:709466. doi: 10.3389/fimmu.2021.709466 (PMC8346206; doi:10.3389/fimmu.2021.709466)
Supplement: Supplementary file 11 [file Table_2.docx]

|  | **RNAseq NF** | | | **qPCR NF** | | |
| --- | --- | --- | --- | --- | --- | --- |
| **Gene** | **Log_2_(CPM) *Lm*** | **Log_2_(FC)** | **FDR** | **RCN *Lm*** | **Log_2_ (FC)** | **P-value** |
|  |  |  |  |  |  |  |
| ***IFNλ1*** | bts | NA | NA | bts | NA | NA |
| ***IFNλ2*** | bts | NA | NA | bts | NA | NA |
| ***TNF*** | 3.14 | 0.26 | 0.81 | bts | NA | NA |
| ***IL-8*** | bts | NA | NA | bts | NA | NA |
| ***IL-1β*** | bts | NA | NA | bts | NA | NA |
| ***IL-6*** | 3.78 | 0.35 | 0.81 | 0.84 | 2.81 | 0.280* |
| ***IL-10*** | bts | NA | NA | bts | NA | NA |
|  |  |  |  |  |  |  |
|  |  |  |  |  |  |  |
|  | **RNAseq F** | | | **qPCR F** | | |
| **Gene** | **Log_2_(CPM) *Lm*** | **Log_2_(FC)** | **FDR** | **RCN *Lm*** | **Log_2_ (FC)** | **P-value** |
|  |  |  |  |  |  |  |
| ***IFNλ1*** | bts | NA | NA | bts | NA | NA |
| ***IFNλ2*** | bts | NA | NA | bts | NA | NA |
| ***TNF*** | -1.04 | -0.24 | 1.00 | bts | NA | NA |
| ***IL-8*** | bts | NA | NA | bts | NA | NA |
| ***IL-1β*** | bts | NA | NA | bts | NA | NA |
| ***IL-6*** | 2.41 | 0.28 | 1.00 | 0.27 | 2.91 | 0.321* |
| ***IL-10*** | bts | NA | NA | bts | NA | NA |

**Supplemental Table 2: RT-qPCR validation of RNAseq (BeWo cells).** A collection of cytokine-coding genes were selected to confirm RNAseq data of infected (*Lm*) and non-infected, fused (F) and non-fused (NF) BeWo cells by RT-qPCR. Data are the average of 3 independent experiments. CPM (counts per million) of *Lm*-infected samples; FC (fold change) compares infected to non-infected samples; FDR (false discovery rate); RCN (relative copy number) of *Lm*-infected samples; SEM (standard error of the mean); BTS (below threshold); NA (not applicable). * = not significant
