# Supplementary material for: Human Placental Trophoblasts Infected by Listeria monocytogenes Undergo a Pro-Inflammatory Switch Associated With Poor Pregnancy Outcomes
Source: Front Immunol. 2021 Jul 23;12:709466. doi: 10.3389/fimmu.2021.709466 (PMC8346206; doi:10.3389/fimmu.2021.709466)
Supplement: Supplementary file 12 [file Table_3.docx]

| **Transcription factor activity** | **Log_2_(FC)** | **FDR** |
| --- | --- | --- |
| Interferon regulatory factor 4 (IRF4) | 3.37 | 1.82E-12 |
| ETS homologous factor (EHF) | 3.05 | 2.24E-11 |
| H2 0 Like Homeobox (HLX) | 2.53 | 1.58E-15 |
| Signal transducer and activator of transcription 5A (STAT5A) | 2.44 | 1.45E-16 |
| THAP domain containing 2 (THAP2) | 2.42 | 4.45E-13 |
| Musculin (MSC) | 2.38 | 1.03E-18 |
| ETS proto-oncogene 1, transcription factor (ETS1) | 2.33 | 4.52E-20 |
| B-cell lymphoma 3 (BCL3) | 2.16 | 5.32E-11 |
| Nuclear factor of activated T-cells 1(NFATC1) | 2.05 | 2.57E-08 |
| Basic leucine zipper ATF-like transcription factor (BATF) | 2.05 | 1.36E-04 |
| Nuclear factor kappa B subunit 1(NFKB1) | 2.00 | 8.94E-12 |
| Nuclear factor kappa B subunit 2 (NFKB2) | 1.97 | 2.00E-08 |
| Early growth response 3 (EGR3) | 1.92 | 7.88E-07 |
| Human immunodeficiency virus type I enhancer binding protein 3 (HIVEP3) | 1.81 | 2.02E-05 |
| SMAD family member 3 (SMAD3) | 1.72 | 4.26E-08 |
| Early growth response 1 (EGR1) | 1.65 | 5.92E-05 |
| REL proto-oncogene, NF-kB subunit (REL) | 1.62 | 3.31E-09 |
| Early growth response 2 (EGR2) | 1.61 | 2.12E-06 |
| HIC ZBTB transcriptional repressor 1 (HIC1) | 1.60 | 3.68E-03 |
| RELB proto-oncogene, NF-kB subunit (RELB) | 1.47 | 6.29E-06 |
| Sp6 transcription factor (SP6) | 1.42 | 5.26E-03 |
| JunB proto-oncogene, AP-1 transcription factor subunit (JUNB) | 1.38 | 3.46E-04 |
| NK3 homeobox 1 (NKX3-1) | 1.36 | 1.06E-06 |
| FOS like 1, AP-1 transcription factor subunit (FOSL1) | 1.33 | 5.53E-06 |
| Jun proto-oncogene, AP-1 transcription factor subunit (JUN) | 1.28 | 4.71E-04 |
| Interferon regulatory factor 7(IRF7) | 1.22 | 1.54E-05 |
| CCAAT/enhancer binding protein beta (CEBPB) | 1.22 | 1.90E-02 |
| Hes family bHLH transcription factor 4 (HES4) | 1.19 | 1.80E-03 |
| ELK3, ETS transcription factor (ELK3) | 1.15 | 1.16E-04 |
| V-myc avian myelocytomatosis viral oncogene homolog (MYC) | 1.14 | 1.98E-04 |
| Human immunodeficiency virus type I enhancer binding protein 2 (HIVEP2) | 1.10 | 1.00E-03 |
| Immediate early response 2 (IER2) | 1.10 | 1.27E-02 |
| MAF bZIP transcription factor F(MAFF) | 1.10 | 2.61E-02 |
| PR/SET domain 1 (PRDM1) | 1.10 | 2.34E-03 |
| Ankyrin repeat domain 1 (ANKRD1) | 1.10 | 2.40E-03 |
| Immediate early response 5 (IER5) | 1.07 | 6.84E-03 |
| Nuclear receptor subfamily 4 group A member 1 (NR4A1) | 1.07 | 1.98E-02 |
| Nuclear receptor subfamily 4 group A member 2 (NR4A2) | 1.07 | 3.68E-03 |
| TATA-box binding protein associated factor 4b (TAF4B) | 1.05 | 5.16E-04 |
| **Transcription co-activators** | **Log_2_(FC)** | **FDR** |
| Mastermind like domain containing 1 (MAMLD1) | 1.22 | 4.65E-04 |
| Proline rich nuclear receptor coactivator (PNRC1) | 1.01 | 4.27E-03 |

**Supplemental Table 3. Transcription factors.** RNAseq analysis was performed to determine how *L. monocytogenes* infection affects the trophoblast transcriptome a 5 h post-infection. Three independent experiments involving 3 different placentas were performed comparing infected to non-infected PHT. We found that among the 12,275 detected PHT genes, 359 were upregulated in response to *L. monocytogenes* infection and, of those, 41 had transcriptional activity.
