# Supplementary material for: Human Placental Trophoblasts Infected by Listeria monocytogenes Undergo a Pro-Inflammatory Switch Associated With Poor Pregnancy Outcomes
Source: Front Immunol. 2021 Jul 23;12:709466. doi: 10.3389/fimmu.2021.709466 (PMC8346206; doi:10.3389/fimmu.2021.709466)
Supplement: Supplementary file 13 [file Table_4.docx]

| **Genes associated with preterm birth/infections of the fetal/placental unit** | **Log_2_(FC)** | **FDR** |
| --- | --- | --- |
| *CCL2* | 2.38 | 1.54E-06 |
| \| \| *CXCL8* \| \| --- \| \| \| --- \| --- \| | 4.17 | 3.81E-19 |
| *F3* | 2.19 | 2.03E-11 |
| *CSF1* | 3.78 | 3.53E-38 |
| *CSF2* | 7.81 | 2.68E-47 |
| *CSF3* | 7.13 | 9.20E-35 |
| *GJB2* | 1.58 | 1.15E-04 |
| *IFNGR2* | 1.51 | 3.49E-07 |
| *IL1A* | 3.36 | 7.53E-15 |
| *IL1B* | 6.53 | 3.03E-64 |
| *IL1RN* | 2.99 | 9.94E-22 |
| *IL10* | 4.70 | 3.80E-11 |
| *IL18* | 1.43 | 1.17E-02 |
| *IL2RA* | 4.02 | 5.18E-30 |
| *IL6R* | 1.17 | 4.08E-05 |
| *IL6* | 3.47 | 5.79E-28 |
| *MMP7* | 1.42 | 9.61E-03 |
| *MMP9* | 1.82 | 1.14E-11 |
| *NFKB1* | 2.00 | 8.94E-12 |
| *PLAUR* | 2.10 | 1.85E-12 |
| *PLAU* | 1.27 | 1.15E-04 |
| *PTGER2* | 2.53 | 2.01E-10 |
| *PTGER4* | 1.23 | 1.37E-04 |
| *PTGS2* | 3.50 | 1.65E-22 |
| *TLR2* | 1.45 | 7.00E-04 |
| *TAP1* | 1.04 | 3.46E-03 |
| *TNF* | 4.78 | 1.69E-45 |
| *NFKB2* | 1.97 | 2.00E-08 |
| *CCL3* | 6.69 | 1.77E-72 |
| *NFKBIA* | 1.66 | 4.53E-06 |
| *NFKBIB* | 1.26 | 2.06E-05 |
| *NFKBIE* | 1.68 | 7.52E-12 |

**Supplemental Table 4: Genes associated with preterm birth.** Gene enrichment analysis using DAVID revealed that 32 upregulated DE genes were associated with preterm birth and infections of the fetal/placental unit. Of these genes, 13 were in the top 50 upregulated DE genes (blue dots, Table 2) and the production of 10 corresponding cytokines was increased in response to infection (green dots). Proteins from genes not labeled with a green dot were not tested.
