# Supplementary material for: Human Placental Trophoblasts Infected by Listeria monocytogenes Undergo a Pro-Inflammatory Switch Associated With Poor Pregnancy Outcomes
Source: Front Immunol. 2021 Jul 23;12:709466. doi: 10.3389/fimmu.2021.709466 (PMC8346206; doi:10.3389/fimmu.2021.709466)
Supplement: Supplementary file 14 [file Table_5.docx]

|  | 5 h Sendai virus | | 10 h Sendai virus | |
| --- | --- | --- | --- | --- |
| **Gene** | RCN | Log_2_(FC) | RCN | Log_2_(FC) |
| ***IFNλ1*** | 39.31 | 8.37 | 14.28 | 6.90 |
| ***IFNλ2*** | 40.84 | 8.42 | 12.56 | 6.72 |
| ***IFNλ3*** | 1.20 | 3.33 | 0.23 | 0.95 |

B

A

|  | 5 h Sendai virus | | 10 h Sendai virus | | 24 h Sendai virus | | 24 h  *L. monocytogenes* | |
| --- | --- | --- | --- | --- | --- | --- | --- | --- |
| **Protein** | pg/ml | Log_2_(FC) | pg/ml | Log_2_(FC) | pg/ml | Log_2_(FC) | pg/ml | Log_2_(FC) |
| **IL-29 (*IFNλ1*)** | 1194.54 | 5.25 | 709.41 | 4.50 | 29452.90 | 9.88 | - | NA |
| **IL-28A (*IFNλ2*)** | 2273.66 | 6.18 | 1578.73 | 5.66 | 65790.37 | 11.04 | - | NA |
| **IL-28B (*IFNλ3*)** | 724.96 | 4.53 | 378.94 | 3.60 | 21428.20 | 9.43 | 155.52 | 2.31 |

**Supplemental Table 5: IFNλ production by Sendai virus- and *L. monocytogenes*-infected PHT**. PHT were infected with Sendai virus, or *L. monocytogenes* for up to 24 h. Cell culture supernatants were collected, centrifuged, and immediately frozen. (A) Cells were lysed with TRIzol and analyzed by RT-qPCR. Data are the average of 2 independent experiments. RCN (relative copy number) in infected samples; FC (fold change) compares infected to non-infected samples at the indicated time points. (**B**) IFNλ (IL-29, IL-28A, IL-28B) protein levels were measured by ELISA at 5 h (n=1), 10 h (n=1), and 24 h (n=2) for Sendai virus- and 24 h (n=1) for *L. monocytogenes-*infected samples. FC (fold change) compares infected samples to the lower limit of detect for the ELISA kit since non-infected samples were below threshold. (-): below detection limit; NA (not applicable).
